# Brain drain or brain circulation? a perspective from Latin American scientists

**DOI:** 10.3389/frma.2026.1852571

**Published:** 2026-07-15

**Authors:** Alina Freire-Fierro, Daniela Pareja-Mejía, Luisa Maria Diele-Viegas, Patricia Castillo-Briceño, Anabel Martinez-Bengochea, Patricia Henriquez-Coronel, J. Francisco Morales, Ana Lucía Bravo-Cazar, Carla Novoa Sepúlveda, Karina Fidanza Rodrigues, Stephanie Vargas Aguilar, Federico Garcia-Yanes, Tatiana Arias

**Affiliations:** 1 Translational Plant Research Group, Faculty of Life Sciences, Universidad Regional Amazónica Ikiam, Tena, Napo, Ecuador; 2 Missouri Botanical Garden, St. Louis, MO, United States; 3 UTCEC Herbarium, Cotopaxi Technical University, Latacunga, Ecuador; 4 O'Connell Laboratory, Department of Biology, School of Humanities and Sciences, Stanford University, Stanford, CA, United States; 5 Grupo de Investigación en Biogeografía y Ecología Espacial, Universidad Regional Amazónica Ikiam, Tena, Ecuador; 6 Laboratório de (Bio) Diversidade no Antropoceno, Universidade Federal da Bahia, Salvador, Bahia, Brazil; 7 Kellogg Ecological Station, Michigan State University, Hickory Corners, Michigan, MI, United States; 8 Global Studies Institute, University of Oregon, Eugene, OR, United States; 9 Equatorial Biome & Ocean Acidification (EBIOAC) Lab, Faculty of Life Sciences & Technologies, Universidad Laica Eloy Alfaro de Manabí (ULEAM), Manta, Manabí, Ecuador; 10 Pacesa Laboratory, Department of Medicine, Institute of Pharmacology and Toxicology, University of Zurich, Zurich, Switzerland; 11 Research Group in Educommunication and Scientific Dissemination for Sustainability (ECODIS), Universidad Laica Eloy Alfaro de Manabí (ULEAM), Manta, Manabí, Ecuador; 12 Universidad Nacional de Educación (UNAE), Chuquipata, Ecuador; 13 Herbarium, University of the West Indies, Campus St. Augustine, Trinidad and Tobago; 14 VALPL Herbarium, University of Playa Ancha, Valparaíso, Chile; 15 Laboratorio de Sistemática y Biogeografía de Plantas, Departamento de Biología, Universidade Estadual de Maringá (UEM), Maringá, Brazil; 16 Department of Molecular Biology, UT Southwestern Medical Center, Dallas, TX, United States; 17 Escuela de Ciencias Biológicas, Grupo de Investigación en Biología para la Conservación, Universidad Pedagógica y Tecnológica de Colombia, Tunja, Colombia; 18 Herbario Nacional de Colombia, Universidad Nacional de Colombia, Bogotá, Colombia; 19 Grupo de Investigación en Biodiversidad, Medio Ambiente y Salud (BIOMAS), Facultad de Ingeniería y Ciencias Aplicadas, Universidad de Las Américas (UDLA), Quito, Ecuador

**Keywords:** brain circulation, Global north, Global South, leadership, scientific diaspora

## Abstract

Though long-standing asymmetries between Global North (GN) and Global South (GS) research systems have shaped the production and circulation of scientific knowledge, a quiet transformation is emerging. Increasing scientific mobility is reframing “brain drain” as brain circulation, highlighting the potential for more reciprocal and equitable exchanges. Scientists from the GS working across borders—the scientific diaspora (SD)—are emerging as key actors in transforming global scientific collaborations. Here, we combine conceptual analysis with survey data from 107 primarily Latin American researchers to examine how structural inequalities and diaspora engagement intersect. Respondents consistently identified interconnected asymmetries, including unequal intellectual recognition, authorship imbalances, and GN dominance in funding and agenda-setting. Publishing systems were also perceived as reinforcing disparities through language barriers and high costs, with women reporting higher levels of structural inequality. Despite these challenges, diaspora scientists are recognized as uniquely positioned to mediate knowledge exchange, expand networks, and foster more equitable collaborations. However, SD transformative potential remains limited by precarious career conditions and weak institutional support. Without structural reform, brain circulation will remain an unrealized promise rather than a pathway toward a more just global scientific system.

## 1 Introduction

The global scientific landscape is shaped by structural inequalities that reflect historical, economic, and geopolitical asymmetries between the Global North (GN) and the Global South (GS). Limited access to funding, infrastructure, and training opportunities have historically driven scientists from GS countries to pursue careers in GN institutions, a process traditionally framed as “brain drain,” implying a unidirectional, permanent loss of human capital that depletes the origin country's capacity (Gaillard and Gaillard, 1997; Sugimoto et al., 2017; Oldac, 2023; Beaty et al., 2024). However, reducing this phenomenon to a simple loss overlooks the dynamics of academic mobility, which represents the physical cross-border movement for training or employment without inherently implying a permanent severing of ties (Robinson-Garcia et al., 2019).

Indeed, many GS scientists residing in the GN maintain connections with their home countries, forming the Scientific Diaspora (SD), which is defined as the community of overseas scientists who actively mobilize knowledge and resources to benefit their home countries (Barre et al., 2003). In this sense, the SD maintains these ties through transnational engagement encompassing remote practices such as joint publications, virtual mentoring, and dual institutional collaborations (Echeverría-King et al., 2022). Therefore, members of the SD, including the authors of this paper, act as bridge-builders, leveraging their acquired capital to catalyze “brain circulation” (Echeverría-King et al., 2022; Bonilla et al., 2023).

Brain circulation holds great potential to reduce global research gaps, by moving beyond “parachute science” (i.e., when GN researchers extract local data without equitable partnership or shared scientific sovereignty; Soares et al., 2025; Marginson, 2016) toward genuine collaborations that prioritize regional expertise and legal processes (Alatas, 2003; Else, 2022; Soares et al., 2025; Haelewaters et al., 2021). Instead of scientists relocating to be reabsorbed by the domestic academic framework in their origin country (Vessuri, 2020), brain circulation depends on a cycle of knowledge and resource exchange that enriches both the host and origin institutions (Meyer, 2001; Echeverría-King et al., 2022). However, this is a two-fold process that requires increased domestic research and education investment, with a strategic approach on prioritizing globally transferable skills to allow their own scientists to lead on critical domestic issues rather than solely following external agendas. Countries such as Bosnia and Herzegovina, the Philippines, Malaysia, Tunisia, Indonesia, and India already have good results with initiatives of skills building tailored to match with the needs of both the domestic market and potential destination countries (World Bank, 2023). In Latin America, researchers can spearhead global initiatives directly from their home institutions, retaining control over research design, funding, and authorship, by streamlining institutional bureaucracy and fostering a culture of regional leadership (Choquez-Millan et al., 2024). On the other hand, Rep. of Korea and Vietnam have programs to maximize the effects of knowledge transfer and engagement with their diaspora to participate in formulating their economic development plans (World Bank, 2023). The alignment between scientific networks and institutional foreign policy directly feeds into science diplomacy, in which international scientific cooperation is leveraged to address shared global challenges, build capacity, and bridge geopolitical divides (Royal Society, 2010; Soler, 2021; Echeverría-King et al., 2023). However, there is also a need to understand the specificities within regions in the GS. Its middle-income countries, represents one such case.

In this article we present insights from research directly from the diaspora, mostly from Latin American middle-income countries of origin and with mixed current destinations, to explore partnership and collaboration models as directly observed and experienced during our academic and research careers as members of the SD. We propose key policy directions for governments and institutions in both the GN and GS to facilitate the transition from brain drain to genuine brain circulation, in which GS institutions would benefit from continuity of trained researchers and strengthened research capacity, and GN institutions would benefit from access to new research questions, environments, and perspectives.

## 2 Methodology

This paper is based on a mixed-methods approach that combines a review of existing literature with survey data collected primarily from Latin American scientists, complemented by collaborative reflections and discussions among the authors. To explore perceptions of structural inequality, decision-making processes, and potential reform in international North–South scientific collaborations, we conducted an anonymous online survey.

The survey was administered in English using Google Forms between November 2025 and January 2026. Participants were recruited through social media platforms (Facebook, Instagram, and LinkedIn), personal and professional networks, email distribution lists, and international scientific organizations such as the Organization for Women in Science for the Developing World (OWSD). Participation was voluntary and anonymous, and responses were collected online through Google Forms. The survey required approximately 20–25 min to complete.

The target population included researchers from both the Global North and Global South, including scientists working in their countries of origin as well as those living and working abroad. Eligible participants were individuals actively engaged in scientific research, higher education, innovation or related scientific activities, regardless of career stage or country of residence. For this study, the scientific diaspora was defined as self-organized, transnational networks of expatriate scientists and scholars who maintain professional connections to their homeland and actively contribute to its scientific, technological, and educational advancement from abroad (Barre et al., 2003). Participants self-identified whether they considered themselves part of the scientific diaspora.

The final sample consisted of 107 valid responses (Table 1). Respondents represented diverse backgrounds, with 79.4% identifying as Latin American/Latino/a/e. Most respondents were affiliated with institutions in the Global South (57%), worked in the life sciences (61.7%), and 54.7% identified as members of the scientific diaspora. The questionnaire included 31 questions (Q1–Q31) organized into four sections. Questions 1–8 collected sociodemographic information, Questions 9–20 assessed perceptions and experiences related to inequalities and power dynamics in international scientific collaboration, Questions 21–26 explored views on decision-making, ownership, and future models of collaboration, and Questions 27–31 consisted of open-ended prompts designed to capture participants' experiences and perspectives.

**Table 1:** Survey design and thematic structure for assessing perceptions of North–South scientific collaboration (*N* = 107).

Section	Items	Format	Content
*Sociodemographic profile* **I**	Q1–Q8	Closed (**categorical**)	*Sociodemographic profile:* gender, age, ethnicity, institutional region, career stage, disciplinary field, N-S collaboration experience, member of scientific diaspora
*Perception of structural inequality in North–South collaboration* **II**	Q9–Q20	Likert scale (1–5)	*Perception of structural inequality in North–South collaboration (12 items):* Unequal intellectual recognition, parachute science, publishing inequalities, inequitable capacity building, structural barriers to equitable partnerships, career and mobility restrictions, the role of the scientific diaspora, one-sided collaborations, task marginalization, authorship and leadership exclusion, and discrimination in global research settings.
*Decision-making, ownership and reform preferences* **III**	Q21–Q26	Closed (single choice)	*Decision-making, ownership, and reform preferences:* location of power, decolonisation, authorship norms, structural barriers, effectiveness of funding models, preferred future model
*Open-ended perceptions* **IV**	Q27–Q31	Open-ended (free text)	*Open-ended perceptions:* Collaboration experience and lessons learned, brain drain vs. brain circulation, one reform to improve equity, mutual misunderstandings GN–GS, meaningful actions for equitable collaboration

Response counts varied slightly across survey items (105–107 responses). Percentages reported in the manuscript were calculated using the number of valid responses for each question. Quantitative data were analyzed descriptively using frequencies and percentages. Open-ended responses were reviewed and grouped into recurring themes related to equity, authorship, mobility, power asymmetries, and the role of scientific diasporas in international collaboration.

Because the survey relied on a convenience sample recruited through professional networks and social media, it may overrepresent researchers already interested in issues of scientific mobility, equity, and North–South collaboration. In addition, given the predominance of respondents identifying as Latin American/Latino/a/e and working in the life sciences, the findings should be interpreted as exploratory and should not be considered representative of all scientists or scientific diasporas worldwide.

## 3 Results

### 3.1 Perceptions on the role of the scientific diaspora in knowledge circulation - survey insights

**Figure 1:**
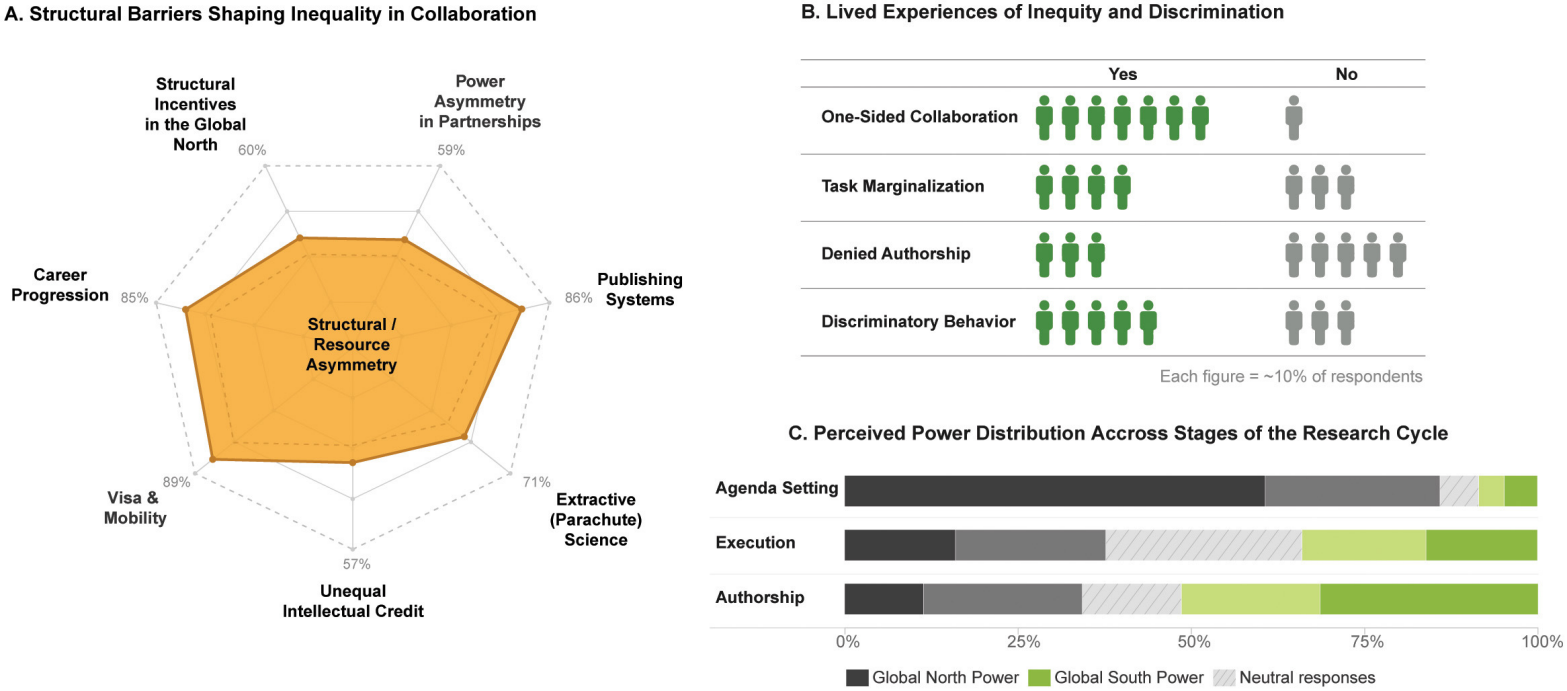
Key survey findings on perceived structural inequalities and lived experiences in North–South scientific collaboration. **(A)**. Percentage of respondents agreeing or strongly agreeing (Likert 4 + 5; *n* = 106–107) with seven structural barrier statements: unequal intellectual credit (Q9), extractive (parachute) science (Q10), publishing systems (Q11), power asymmetry in partnerships (Q12), structural incentives in the Global North (Q13), career progression (Q14), and visa and mobility constraints (Q15). **(B)**. Proportion of respondents reporting agreement (Agree + Strongly Agree, green) vs. disagreement (Disagree + Strongly Disagree, gray) with four lived experience statements: one-sided collaboration (Q17), task marginalization (Q18), denied authorship (Q19), and discriminatory behavior (Q20); each icon represents −10% of respondents (*n* = 105–107). **(C)**. Perceived power distribution across three research stages — agenda setting (Q11), execution (Q18), and authorship (Q19) — from agreement with each item (5-point Likert; *n* = 105–107). Global North power (dark gray = strongly agree, light gray = agree), neutral (hatched), Global South power (light green = disagree, dark green = strongly disagree).

Our survey sample comprised researchers primarily affiliated with Global South (GS) institutions (57%), with smaller proportions from the Global North (GN; 29%) and from dual affiliations (10.3%). Respondents were predominantly female (57.9%) and of Latin American/Latino background (79.4%); most were aged 41–50 years (38.3%) and worked in the life sciences (61.7%). Across items addressing unequal recognition, parachute science, publishing asymmetries, and visa restrictions responses were strongly skewed toward agreement, indicating broad consensus that structural imbalances systematically impede equitable knowledge circulation between GN and GS institutions (Figure 1; Supplementary Material Figure 1).

Perceptions of structural and systemic inequities were widespread across multiple dimensions (Figure 1A). The strongest signals were unequal intellectual and academic benefits (Q9: 57.0%, *n* = 61/107) and authorship asymmetries, which disadvantaged GS researchers (Q20: 50.9%, *n* = 54/106). Female respondents reported higher perceived structural inequality than men, particularly on publishing access (Q11: women 91.9% vs. men 77.8%) and parachute science dynamics (Q10: women 75.4% vs. men 64.4%). Parachute science practices were commonly linked to one-sided flows of data and samples from South to North (Figure 1B).

A second pattern centered on agenda-setting. Respondents pointed to institutional incentives in the GN (Q13: 60.4%, *n* = 64/106) alongside career progression barriers for GS researchers (Q14: 85.0%, *n*=91/107), suggesting that extractive practices, authorship marginalization, funding inequalities, and epistemic hierarchies are widely perceived as interconnected (Figure 1C). Publishing systems were a recurring concern (Q11: 85.9%, *n* = 92/107): respondents described them as favoring English-language outlets, imposing exorbitant publication fees, and applying editorial standards rooted in Northern academic traditions (Supplementary Material 1). Most respondents perceived decision-making authority to be concentrated in funding bodies and high-prestige institutions, with only a small minority viewing power as fairly negotiated (Supplementary Material 1).

Decolonization of science was considered valuable but difficult to implement by 48.6% of respondents (*n* = 52/107, Q22), while 32.7% (*n* = 35/107, Q22) considered it essential and overdue. Gender differences were evident: women were more likely than men to call it essential (40.3%, *n* = 25/62 vs. 22.2%, *n* = 10/45, Q22), whereas men more often described it as politically divisive (22.2%, *n* = 10/45, vs. 9.7%, *n* = 6/62, Q22). Just over half of the respondents 54.2% of respondents (*n* = 58/107, Q23) preferred co-first authorship or shared-leadership models over approaches based on individual contribution.

At the level of lived experience, respondents identified personal structural career constraints — unstable employment, restricted funding access, and limited leadership prospects — as key factors undermining their capacity to negotiate equitable collaborative conditions (Figure 1B). The absence of institutional mechanisms for diaspora engagement, reintegration, and systematic support emerged as a critical gap limiting the diaspora's transformative potential. Structural power imbalances were identified as the primary barrier to equitable partnerships, followed by bureaucratic constraints and institutional instability in GS institutions. Reformed North–South partnerships with shared leadership emerged as the most widely preferred model for future collaboration (Supplementary Material 1).

Taken together, these data describe a research community that is structurally constrained yet analytical about its own position. Respondents portrayed a system in which Southern partners face restricted career opportunities, extractive collaboration dynamics, and a publishing infrastructure that privileges northern agendas, all this while navigating practical barriers including limited mobility and visa access. Two findings stand out. First, the most widely preferred collaboration model was reformed North–South partnerships with shared leadership, chosen by 57.0% of respondents (*n* = 61/107, Q26), with diaspora-anchored networks the second choice (25.2%, *n* = 27/107); together, these two diaspora-engaging models accounted for 82.2% of preferences (*n* = 88/107), positioning the diaspora as an active instrument of change rather than a passive outcome. Second, the broad support for co-first authorship and shared-leadership models indicates that the reforms proposed here have backing from the people they would most directly affect.

Regarding open-ended questions, respondents identified eight categories of proposed change, dominated by four. The most frequently raised was funding reform; respondents argued that current systems concentrate financial control in the GN and called for direct funding to GS institutions, shared budget control, more investment in low- and middle-income countries, and long-term capacity-building. Second was shared leadership, objection to Northern institutions setting agendas and budgets before Southern partners were involved, with calls for co-leadership from the outset. Third was fair authorship and credit, as local collaborators who provide permits, field access, and local knowledge are often under-recognized in publications. Fourth was reducing bureaucratic barriers such as permits, sample-transfer rules, and travel red tape. Overall, respondents framed North–South inequity as a governance problem rather than one of individual goodwill (Table 1, Supplementary Material 1).

## 4 Discussion

### 4.1 The diaspora as a strategic asset: collective strengths

Our survey results (Figure 1; Supplementary Material 1) demonstrate that structural and systemic inequities are neither marginal nor anecdotal, but instead persistent across multiple dimensions of North–South scientific collaboration. More than 80% of respondents agreed or strongly agreed (Likert 4–5) with three statements: publishing systems bias (Q11: 85.9 %, *n* = 92/107), career progression barriers for GS researchers (Q14: 85.0%, *n* = 91/107), and visa and mobility barriers (Q15: 88.8%, *n* = 95/107). Levels of agreement were lower, though still substantial, for extractive “parachute science” practices (Q10: 70.8%, *n* = 75/106), institutional incentives in the GN (Q13: 60.3%, 64/106), capacity building and power asymmetries (Q12: 59.4%, 63/106), and unequal intellectual credit (Q9: 57.0%, *n* = 61/107). Overall, these results reveal a broadly shared set of experiences among Latin American scientists and provide empirical support for persistent structural inequalities in international research collaborations. Notably, only a small proportion of respondents perceived power dynamics within current collaborations as equitably negotiated, reinforcing the interpretation that these inequities reflect systemic shortcomings rather than isolated instances. Concurrently, the majority preference for shared leadership models in North–South partnerships (Q9: 57%) indicates that more equitable alternatives are not only desirable but also broadly supported within the community.

In the following sections, we structure our discussion around four collective strengths of the scientific diaspora (SD), grounding each in both the empirical trends identified in our survey and the existing literature.

*1. Knowledge & Technology Transfer-* Old dominant unidirectional models, where protocols are produced in the GN and exported for passive adoption, often reinforce dependency and reproduce forms of intellectual extraction — defined here as the appropriation of knowledge generated in the GS without reciprocal recognition or benefit (UNCTAD, 2014). In contrast, genuine brain circulation, understood as the recursive and bidirectional movement of knowledge across borders (Saxenian, 2005), entails a process grounded in contextual translation and collaborative embedding rather than simple transfer. That distinction is evidenced by our survey respondents, who identified unequal intellectual and academic benefits (Q9) and authorship asymmetries (Q19) as among the strongest signals of structural inequity (Figures 1A, C) — suggesting that even when knowledge apparently moves South–North–South, recognition and credit remain disproportionately concentrated in the North. This gap between movement and equity positions the SD as a critical catalyst: SD members help transform linear transfers into processes of equitable co-creation, challenging prevailing assumptions about the direction and ownership of innovation flows (UNCTAD, 2014; Echeverría-King et al., 2022). Thus, our findings, highlight that at least for Latin American SD from middle-income countries, the path is still building to reach the reciprocal partnerships (e.g., Klebel and Ross-Hellauer, 2023; Djaafara et al., 2025; Turba et al., 2026), and horizontal South-South collaborations (Enwere et al., 2025), as reported for other nations.

Still, the catalytic role of SD members in the process to arrive to South-North partnerships with local experts taking leadership roles, is illustrated by three examples. The first concerns the adaptation of biotechnological methodologies for territory-specific initiatives. Direct implementation of standardized protocols proved ineffective, requiring fundamental re-engineering of workflows to align with local ecological knowledge and community governance structures. In this case, SD members served as the decisive catalyst — making adaptation workable and reframing feasibility from a purely technical requirement into a socio-ecological achievement that depended on, and was validated by, local legitimacy (Echeverría-King et al., 2022). The second example concerns the implementation of international quality systems, such as Good Manufacturing Practice (GMP), where sustainable compliance emerged only through iterative adaptation to local regulatory frameworks. Here again, SD members played a bridging role, translating normative international standards into locally operable procedures and negotiating between institutional requirements and on-the-ground constraints — a process that would not have been viable without their dual embeddedness in both global and local knowledge environments. The third example concerns the southern diaspora during the COVID-19 crisis. Beyond remittances and the mobilization of supplies, a new generation of diaspora professionals acted as social entrepreneurs, using collaborative, non-profit models to build relationships with in-country counterparts and deploying telemedicine such as teleradiology and telepsychiatry to extend specialist advice and support clinical care. Here again, SD members served as a catalyst, brokering collaborations among diaspora-led organizations, local NGOs and professional associations that linked external expertise to home-country institutions (Jindal et al., 2022).

Taken together, these cases show that GS capacity is not deficient but differently configured, excelling in adaptive innovation that standard evaluation metrics tend to overlook (Kraemer-Mbula and Wamae, 2010; French et al., 2024). The cases underscore how the absence of actors fluent in both international scientific standards and local institutional logics constrains effective implementation. Combining advanced technical training from GN institutions with deep familiarity within their home territories, the SD act as translators, mediators, and institutional brokers (Tejada et al., 2010; Mogaji, 2025). By interpreting the underlying rationales of quality and safety, SD enables principled adaptation without undermining core objectives. However, diaspora expertise remains underutilized due to institutional rigidity and insufficient funding for the translational labor they are uniquely positioned to perform (French et al., 2024; Mogaji, 2025). These dynamics are corroborated by our survey data, which point to career precarity, funding barriers, and limited leadership opportunities as key constraints on diaspora-led translation and institutional change. Most respondents (57 % *n* = 6/107, Q26) identified shared-leadership North–South partnerships as the preferred pathway forward (Figure 1).

*2. Network Access & Internationalization-* Diaspora scientists could rebalance current GN/GS economic and infrastructure imbalances through professional trajectories that span continents, as researchers navigate Northern systems while maintaining deep-rooted ties to GS institutions (Meyer and Brown, 1999; Kuznetsov, 2006; Beaty et al., 2024). This dual positioning places them as strategic mediators capable of rebalancing the internationalization process (Meyer and Brown, 1999; Kuznetsov, 2006; Soares et al., 2025). By moving beyond the passive adoption of established institutional pathways, diaspora scientists actively construct alternative infrastructures (Kuznetsov, 2006). They leverage their dual affiliations to create new network models, such as South–South collaborations and women or indigenous people-led initiatives, prioritizing relational mentorship over traditional hierarchical growth (Kuznetsov, 2006; Gamlen, 2006; Asim et al., 2023).

Successful examples of long-term networks lead from the GS, include the Amazon Tree Diversity Network^1^, Macrolatinos^2^, and the Latin American Society for Developmental Biology^3^ demonstrating the power of regional resource-sharing and targeted brain circulation (Pedone and Izquierdo, 2018; Maldonado et al., 2002; Echeverría-King et al., 2022). Similarly, the Brazilian “Flora e Funga do Brasil.”^4^ The project, aiming to describe all Brazilian flora and fungal species, is led by Brazilian researchers, including members of the SD, with collaborations worldwide (see Supplementary Table 2 for other examples). This strategic leverage ensures that research questions are increasingly defined from a Southern perspective, even when engaging global partners (Kuznetsov, 2006). By coordinating multi-continental teams that bridge high-resolution genomics with local community engagement — as in, for example, Ferns of Colombia^5^— Global South scientists ensure that regional expertise dictates the trajectory of collaborative work (Kuznetsov, 2006; Gamlen, 2006; Stefanoudis et al., 2021; Else, 2022). These examples represent direct challenges to “parachute science.”

The persistence of ethics dumping (Else, 2022) and unequal intellectual relations is driven by financing, assessment, and publishing regimes that prioritize speed, individual status, and extractive outputs over long-term reciprocal collaboration (Haelewaters et al., 2021; Stefanoudis et al., 2021). Respondents of our survey emphasized that these interactions reinforce epistemic hierarchies by determining who is positioned as labor and who is recognized as a legitimate knowledge producer (Smith et al., 2014). English-language publishing norms, high publication fees, and editorial standards rooted in Northern academic traditions were identified as mechanisms that undervalue research with strong local or contextual relevance, limiting GS visibility and influence (Figure 1; Supplementary Material 1). The GS has already demonstrated viable alternatives to Northern-dominated publishing through diamond open access platforms such as SciELO, Redalyc, AmeliCA, AJOL, and regional repositories across Africa and Asia. These scholar-led initiatives support multilingual, equitable, and low-cost knowledge dissemination, challenging both APC-based and English-dominated publishing models (Beigel, 2026; Humberto et al., 2025).

The diaspora's role extends to dismantling financial and gatekeeping barriers that hinder GS visibility by contributing to the global scientific dialogue (Klebel and Ross-Hellauer, 2023; Borrego, 2023; Turba et al., 2026). This translational labor facilitates long-term capacity building, knowledge democratization, visibility, and co-authorship, which are necessary for professional advancement (Gaillard and Gaillard, 1997; Meyer and Brown, 1999; Kuznetsov, 2006). Ultimately, by transforming individual mobility into a collective strategic asset, the diaspora contributes to an internationalization model with shared leadership and equitable resource access (Marginson, 2016; Soares et al., 2025). By facilitating training in cutting-edge laboratories and opening pathways to doctoral programs, SD provides GS scholars with access to analytical techniques that may be unavailable in their home countries (Kuznetsov, 2006). These diasporas, hence, maintain active collaborative links while training the next generation of scientists (Gaillard and Gaillard, 1997; Meyer and Brown, 1999).

Through sustained transnational engagement, diaspora networks prioritize local research agendas while maintaining global connectivity. In doing so, SD can mediate scientific exchange while contesting unequal funding structures and narrow definitions of expertise, contributing to a more inclusive and socially grounded research system (Turba et al., 2026). Ultimately, the diaspora moves internationalization beyond quantitative metrics toward a relational process (Gamlen, 2006; Soares et al., 2025).

*3. Capacity Building & Mentorship-* Diaspora scientists often face persistent structural barriers, including professional insecurity, dependence on external funding, linguistic and bureaucratic challenges, and limited institutional recognition. Such barriers generate precarity and constrain their long-term contributions, exposing systemic weaknesses in international academic systems and highlighting the absence of coordinated mechanisms for support, reintegration, and collaboration. As reflected in our survey (Figure 1; Supplementary Material 1), such constraints limit the transformative potential of scientific mobility and underscore the need for models that prioritize capacity building and mentorship.

Despite these challenges, the SD plays a critical role in strengthening research capacity across the GS in strategic, horizontal, and sustainable ways (Echeverría-King et al., 2022; Bonilla et al., 2023). The most lasting impact emerges from the intentional construction of ecosystems centered on training, mentorship, and scientific leadership. Because capacity building driven by the SD extends far beyond technical knowledge transfer, it requires sustained investment in people, institutions, and local scientific cultures (Confraria, 2019). This process includes supporting early-career researchers, including thorough structured career guidance, co-authorship, and the cultivation of transversal skills such as science communication, policy engagement, and academic leadership. Such mentorship is most effective when it explicitly acknowledges structural inequalities (Deanna et al., 2022), values indigenous knowledge systems, and prioritizes scientific autonomy (Gewin, 2023; Hird et al., 2023). Furthermore, the SD bridges the gap between GS researchers and international funding calls, research infrastructure, and global networks (Bonilla et al., 2023). However, to yield genuine capacity, these connections must be founded on principles of equity, shared leadership, and mutual accountability (Graves et al., 2022), meaning co-designing research agendas and implementing fair authorship practices that recognize intellectual contributions across all research stages (Haelewaters et al., 2021).

A central contribution of the SD lies in fostering the new generation of leaders committed to social impact, inclusion, and systemic transformation (Graves et al., 2022). Intergenerational mentorship programs and peer-support networks are essential to addressing the isolation, precarity, and discrimination that disproportionally affect GS scientists navigating transnational careers. In this sense, support structures strengthen collective scientific resilience while improving individual wellbeing, as in the case of The Organization for Women in Science for the Developing World^6^, which has demonstrated positive outcomes (Blowers et al., 2025).

Future collaboration models should rest on three pillars. The first is equity in leadership: joint strategic decision-making in which both partners shape direction. The second is operationalisation: moving from principle to practice by co-designing projects from the ground up, sharing financial control, and ensuring equitable authorship and recognition. The third is a redefinition of institutional roles, in which international NGOs act as facilitators and local NGOs as territorial leaders, building local capacity over the medium and long term. Ultimately, capacity building through the SD is best understood as a relational, ongoing process rather than a singular intervention — most effective when aligned with institutional policies, national science and technology agendas, and regional strategies for scientific development (Echeverría-King et al., 2022). By integrating mentorship with advocacy and network access, diaspora scientists contribute to more just, diverse, and resilient scientific systems.

*4. Science Diplomacy & Advocacy*- The SD plays a key role in science diplomacy, by influencing science policy decisions through the production of contextualized evidence, participation in advisory spaces, and the translation of knowledge across political, cultural, and scientific systems. Examples for these policies have been observed in Colombia^7^, Ecuador (Feyen et al., 2016; Bonilla et al., 2021), and Mexico (Pedone and Izquierdo, 2018). Diplomatic and advocacy roles of the SD derive from dual credibility across systems: international expertise and networks enhance influence in policy debates in countries of origin, while contextual knowledge of local constraints informs more effective cooperation in host countries, enabling advocacy and agenda-shaping in both directions. Our data indicates that 24.3% of respondents (*n* = 26/107, Q21) perceived power in North–South collaborations to reside more in individuals than in institutions, lending support to the view that well-positioned diaspora scientists can exercise meaningful influence despite structural asymmetries (Supplementary Material 1).

National scholarship programs can foster science diplomacy by enabling targeted international participation. For example, Spain's FPU program, for instance, reserves a small quota for non-EU nationalities, facilitating integration into less internationalized institutions and allowing foreign scholars to act as transnational connectors through their research (BOE^8^). However, the FPU calls have changed eligibility criteria requiring valid residency permits during economic crises. This change illustrates how migratory status has been used to structure internationalization. This pattern positions foreign researchers as strategic interlocutors, yet their impact is frequently undermined by broader structural hurdles. Specifically, mobility and visa restrictions worsen these disparities by restricting attendance at conferences, training sessions, and decision-making venues, where the very networks and visibility these programs aim to foster are developed. The diplomatic potential of the SD is contingent on continued embeddedness in both contexts. When engagement remains reciprocal and informed by local priorities, diaspora actors can mediate collaboration in ways that recalibrate asymmetries rather than reproduce them.

**Figure 2:**
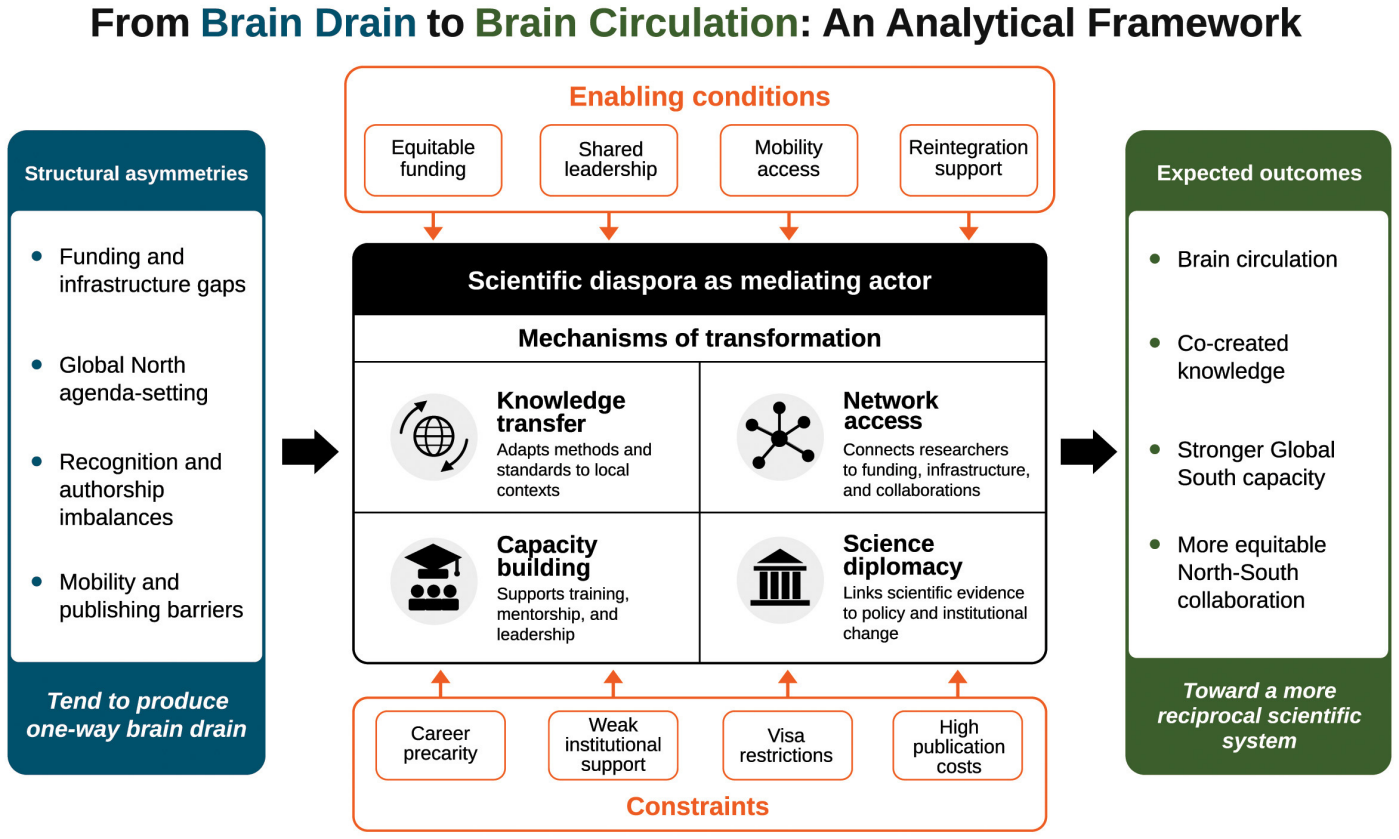
Conceptual framework illustrating the role of the Scientific Diaspora (SD) in transforming structural inequities in global science into equitable knowledge co-creation. The left panel depicts the current challenges faced by the GS, including data extraction, unequal collaboration, and structural barriers that drive brain drain. The central panel positions the SD as catalysts of change, leveraging their dual embeddedness in both Global North and Global South contexts to facilitate network access, technology transfer, and the integration of contextual Southern knowledge. The right panel represents the outcome of SD-mediated brain circulation: a recursive model of knowledge co-creation characterized by equitable collaboration, shared leadership, capacity building, and science diplomacy.

Considering the survey evidence, the literature, and our collective reflections, we propose a set of key policy directions to reduce persistent inequalities between the GN and GS (Table 2, Figure 2). The persistent “brain drain” in the GS is often less a choice than a systemic consequence of the absence of reintegration structures. When institutional support and research continuity are lacking, the potential for circular mobility is lost to one-way migration. Engaging SD offers a concrete pathway to reduce inequalities in GS scientific research by mitigating brain drain and fostering more reciprocal brain-circulation processes between GS and GN. Through the critical work of intercultural mediation and institutional alignment, diaspora scientists can bridge systems and expectations, mitigate regional inequalities, and ultimately offset the loss of human capital through a dynamic and reciprocal exchange of knowledge. In doing so, SD can effectively engage both governmental actors in the GS and institutional and individual peers in the GN, fostering more equitable forms of scientific diplomacy.

**Table 2:** Normative policy synthesis to promote equitable brain circulation between the Global North and Global South.

Policy domain	Global North—suggested actions	Global South—suggested actions
*Mobility and access*	Reduce visa barriers, ensure safe mobility for GS researchers, and expand equitable international exchange and training programs.	Facilitate participation of local researchers in international collaborations, ensure safe mobility for GN researchers, and promote bilateral mobility initiatives.
*Funding and research infrastructure*	Expand funding mechanisms supporting GN–GS collaborations and share research infrastructure.	Increase national investment in research and innovation and ensure access to equipment and infrastructure from international collaborations.
*Equity in research collaboration*	Ensure meaningful participation of GS scientists in research design, funding access, data analysis, authorship, and leadership roles.	Promote institutional participation in international projects and reduce bureaucratic barriers to scientific collaboration.
*Scientific publishing and knowledge access*	Promote fair authorship practices, diversify editorial boards, reduce peer-review bias, and improve access to paywalled publications.	Recognize regional journals, support multilingual publishing, and provide funding to cover publication fees (APCs).
*Capacity building and mentorship*	Expand international scholarships and mentorship programs and provide opportunities for GS researchers to act as advisors and research leaders	Create reintegration mechanisms, stable career pathways, and leadership opportunities for scientists trained abroad.
*Science diplomacy and global leadership*	Encourage participation of GS scientists in global scientific governance, editorial leadership, and international decision-making spaces.	Promote science diplomacy initiatives and national policies strengthening international research networks.

The SD potential to drive institutional change rests on four interconnected capacities: transferring knowledge and technology across systems, building and sustaining international networks, mentoring the next generation of researchers, and acting as advocates at the science-policy interface. Table 2 should therefore be read as a normative policy synthesis, rather than as a direct empirical result. It translates these capacities into reform guidelines spanning six domains, grounded in our survey findings, the broader literature and the firsthand experience of a research team that is itself part of the diaspora.

### 4.2 Study limitations and future directions

Because our survey respondents are concentrated in Latin American life sciences, future work should broaden the geographic and disciplinary scope to test whether the patterns described here extend to other regions and disciplines of the GS. As well as other metrics and approaches such as the income level of the nations, or the Global Innovation Index (GII) must be needed for a better understanding of GS key aspects that can be factors with stronger impact than geographic closeness. A key limitation of our study is that it only captures a single moment in diaspora scientists' careers rather than their full trajectories. Future research should follow the same individuals over time, enabling examination of how career perceptions, mobility decisions and structural barriers shift across career stages. Combining traceability surveys with external data sources — bibliometrics, funding records, authorship audits, collaboration outcomes — would allow researchers to test whether the perceptions captured here correspond to measurable patterns in scientific practice. Most urgently, studies that assess the effectiveness of specific interventions — reintegration programs, bilateral mobility schemes, co-leadership funding requirements — would provide the empirical grounding that reform proposals currently lack.

## 5 Conclusions

Realizing the potential of brain circulation, however, cannot depend on individual agency alone. It requires institutions in both the GN and GS to move from stated commitments to enforceable ones: equitable authorship policies, structural support for diaspora engagement, and shared governance of research agendas. The scientific diaspora cannot restructure global science by itself. But given the right institutional conditions, brain circulation can become the norm, and with it, a global scientific system that is more equitable not only in its outputs, but in how it decides what gets produced and who gets credit for it. The “brain drain” frame captures outflow but misses function. Diaspora scientists are not simply lost to their home systems, they operate as connectors, translators, and intermediaries between research systems that rarely speak directly to each other. Our data show that the community recognizes this and is ready to formalize it. What remains is the institutional will to match that readiness.

## Data Availability

The original contributions presented in the study are included in the article/Supplementary material, further inquiries can be directed to the corresponding authors.

## Footnotes

1
https://sites.google.com/naturalis.nl/amazon-tree-diversity-network/homepage

2
https://www.macrolatinos.net/

3
http://lasdb-development.org/

4
https://reflora.jbrj.gov.br/reflora/PrincipalUC/PrincipalUC.do;jsessionid=6D0E6E82495C31CB40867224FBCA5949

5
https://www.fernsofcolombia.com/

6
https://owsd.net/

7
https://repositorio.minciencias.gov.co/handle/20.500.14143/50811

8
https://www.boe.es
