# Supplementary material for: Brain drain or brain circulation? a perspective from Latin American scientists
Source: Front Res Metr Anal. 2026 Jul 15;11:1852571. doi: 10.3389/frma.2026.1852571 (PMC13415780; doi:10.3389/frma.2026.1852571)
Supplement: Supplementary file 2 [file Data_Sheet_1.pdf]

Survey: *The Unspoken Rules of Global Scientific Diaspora*

TOC

Section 1 - About you (Social-demographics) **2**

Q1 Gender 2

Q3 Ethnicity 2

Q4 Where is your main scientific affilation located? 3

Q5 Career Stage 3

Q6 Field of research 3

Q7 Do you currently collaborate with a country from the opposite hemisphere (meaning Global North or Global South) of your own? 4

Q8 Are you part of the scientific diaspora? 4

Section 2 – Realities, Inequalities, and Power Dynamics in Collaboration (Q9-Q20) **5**

Structural & Systemic Issues (Q9-Q16) **5**

Q9. Benefits in North–South collaborations often do not reflect equal intellectual partnership 5

Q10. "Parachute science" remains a significant problem in global research. 5

Q11. High-impact publishing systems privilege Northern research agendas and methodologies 5

Q12. The concept of "capacity building" is often used in ways that reinforce unequal power dynamics. ... 6

Q13. Structural incentives in the Global North discourage truly equitable partnership. 6

Q14. Being based in the Global South restricts scientific career opportunities 6

Q15. Researchers from the Global South face significant mobility and visa barriers 7

Q16. Diaspora scientists often act as more effective bridges for equity than institution-to-institution agreements 7

Lived Experiences (Q17 – Q20) **7**

Q17. Some collaborations feel one-sided: data/resources flow South → North, while credit stays North. .. 7

Q18. I have been assigned basic or repetitive tasks instead of meaningful scientific responsibilities 8

Q19. I have been denied authorship or leadership opportunities that I felt I deserved. 8

Q20. I have experienced or witnessed subtle discriminatory behavior in global research settings 8

Section 3 – Decision-Making, Ownership, and the Future (Q21- Q26) **9**

Q21. Who typically holds the real power in Global North–South collaborations? 9

Q 22. The concept of "decolonizing science" is 9

Q 23. Authorship in high-impact papers should primarily reflect: 9

Q 24. The biggest barrier to equitable North–South collaboration is 10

Q 25. How effective are current international funding models (EU, NIH, Wellcome, NSF)? 10

Q 26. The most promising future model for global collaboration: 10

Section 4 – Open Questions (Q27-Q31) **11**

Q27 Describe one experience—positive or negative—that best illustrates your view of North–South collaboration. What was the key lesson? 11

Q28 Do you see "brain drain" as primarily a loss, or does "brain circulation" create overlooked opportunities? Explain. 16

Q29 If you could change one rule or norm in global science to make collaborations more equitable, what would it be and why? 21

Q30 What is one thing researchers from the Global North often misunderstand about working with Global South partners? And vice versa? 26

Q31 What do you think are the most meaningful actions—at the individual or institutional level—that could help build more equitable and respectful relationships in global scientific collaboration? 31

Section 1 - About you (Social-demographics)

Q1 Gender

Gender
107 responses

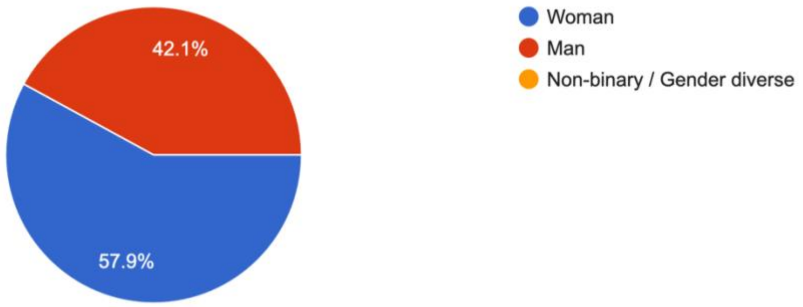

Q2 Age

Age (years)
105 responses

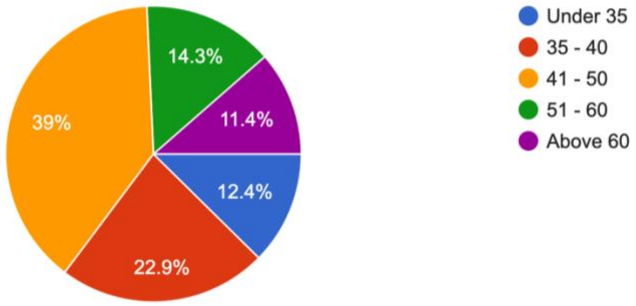

Q3 Ethnicity

Ethnicity (select the option that best reflects how you identify):
107 responses

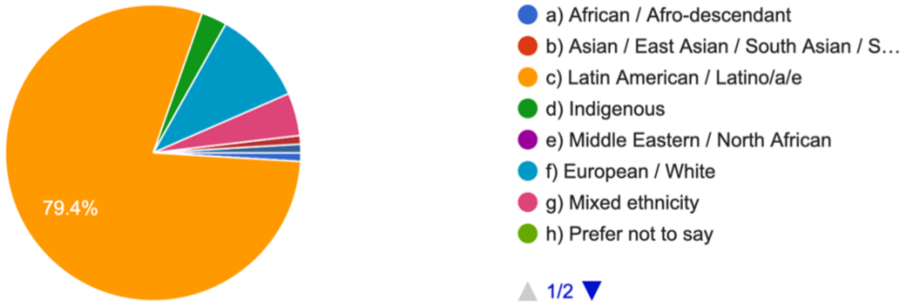

Q4 Where is your main scientific affilation located?

Where is your main scientific affiliation located?

107 responses

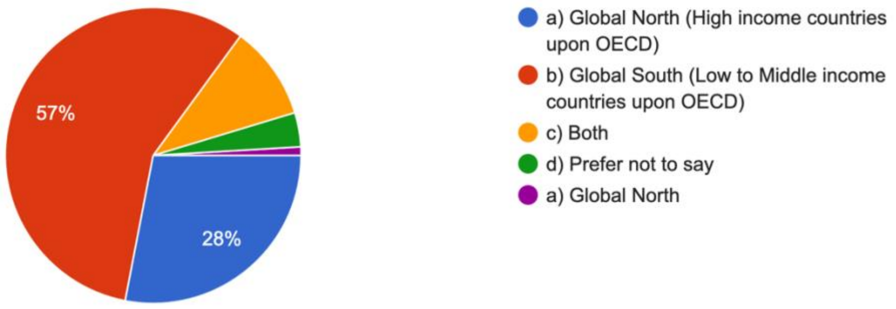

Q5 Career Stage

Career stage:

106 responses

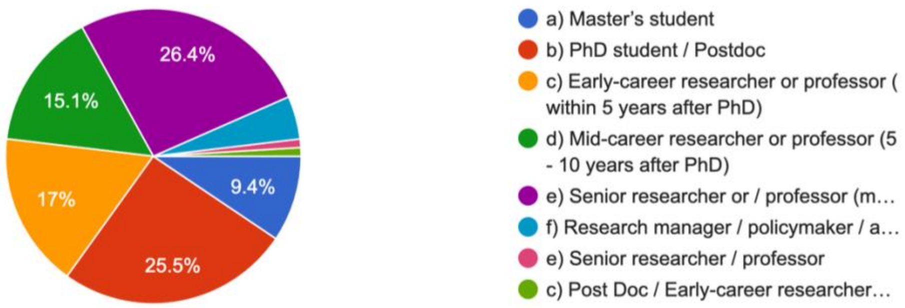

Q6 Field of research

Field of research:

107 responses

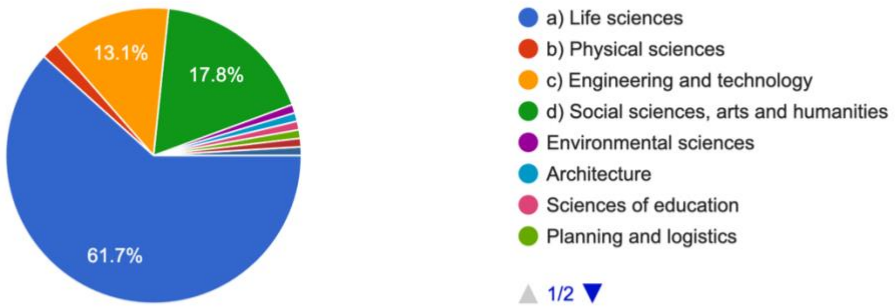

Q7 Do you currently collaborate with a country from the opposite hemisphere (meaning Global North or Global South) of your own?

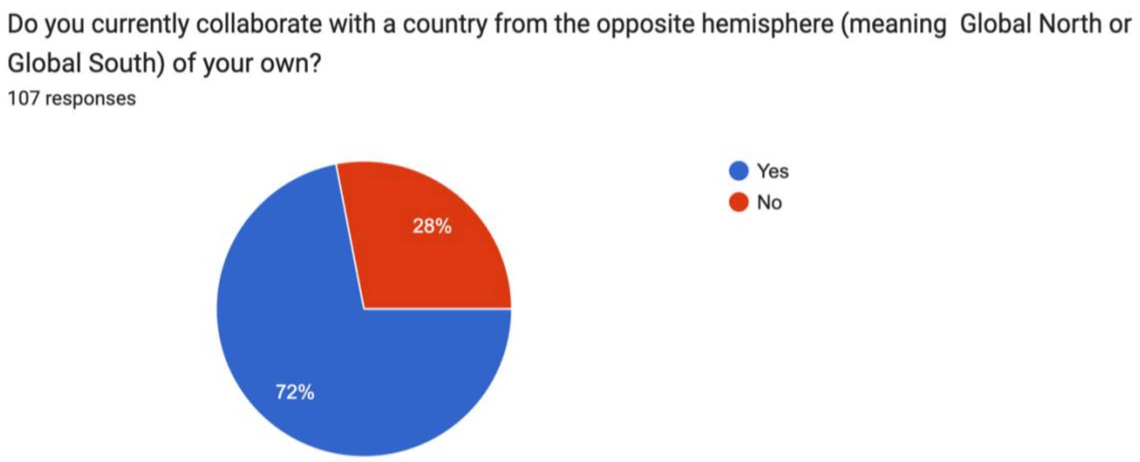

Q8 Are you part of the scientific diaspora?

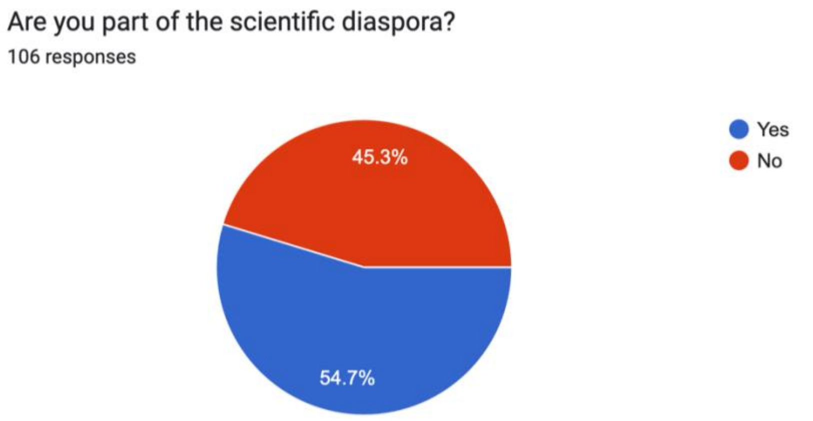

Section 2 – Realities, Inequalities, and Power Dynamics in Collaboration (Q9-Q20)

Likert`s sacle *(1–5): Strongly Disagree → Strongly Agree*

Structural & Systemic Issues (Q9-Q16)

Q9. Benefits in North–South collaborations often do not reflect equal intellectual partnership

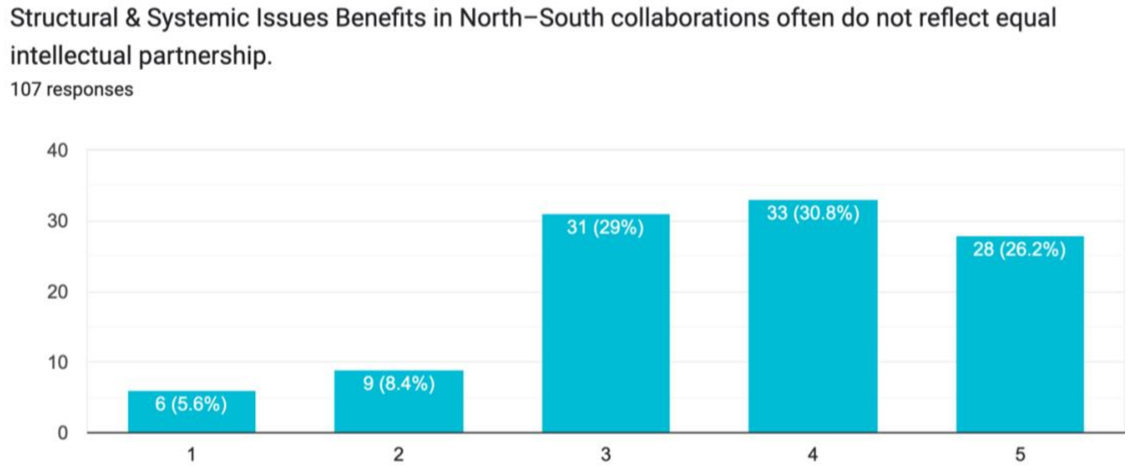

Q10. “Parachute science” remains a significant problem in global research.

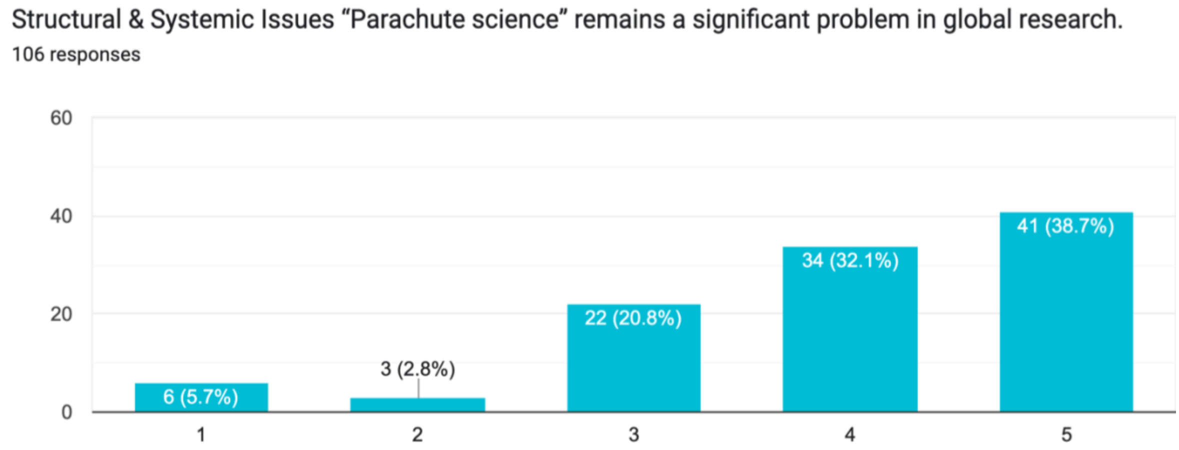

Q11. High-impact publishing systems privilege Northern research agendas and methodologies.

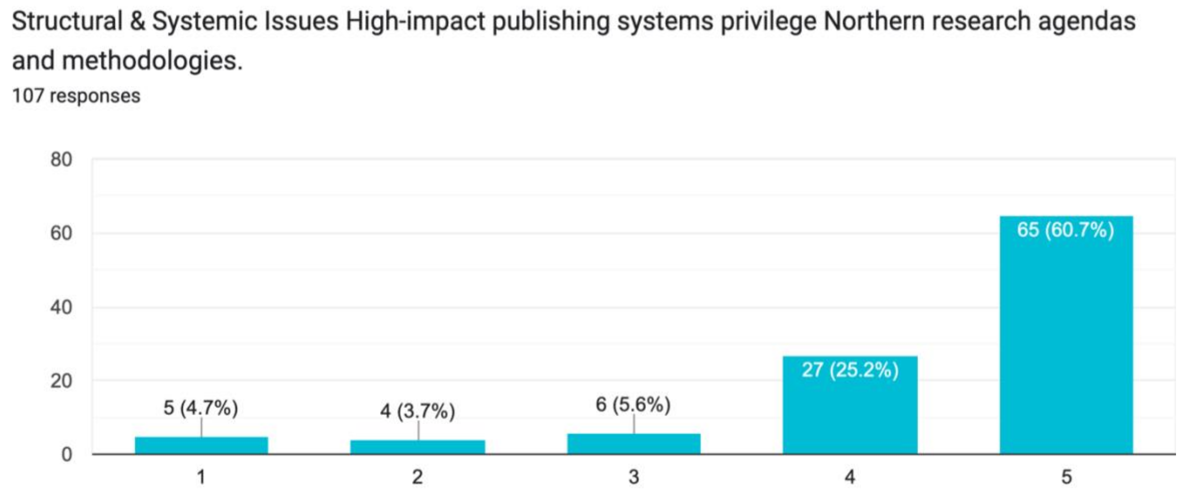

Q12. The concept of "capacity building" is often used in ways that reinforce unequal power dynamics.

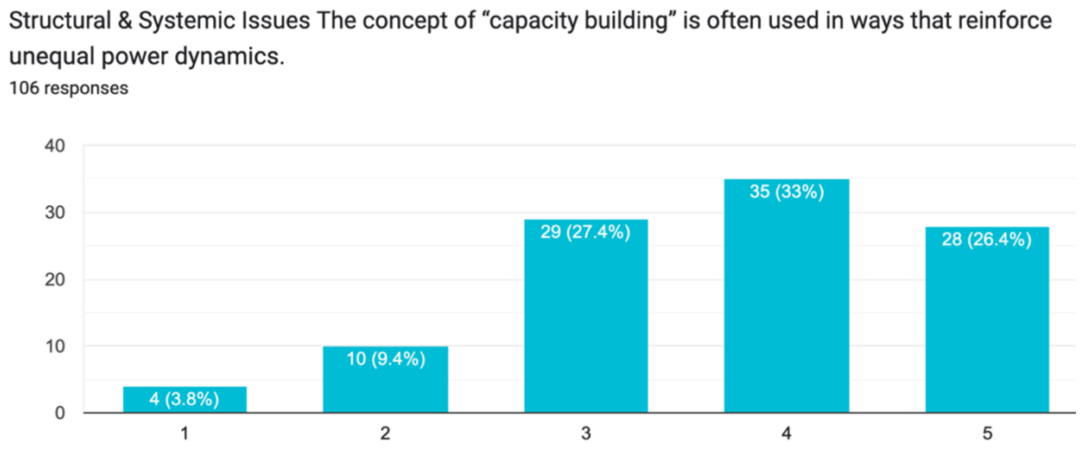

Q13. Structural incentives in the Global North discourage truly equitable partnership.

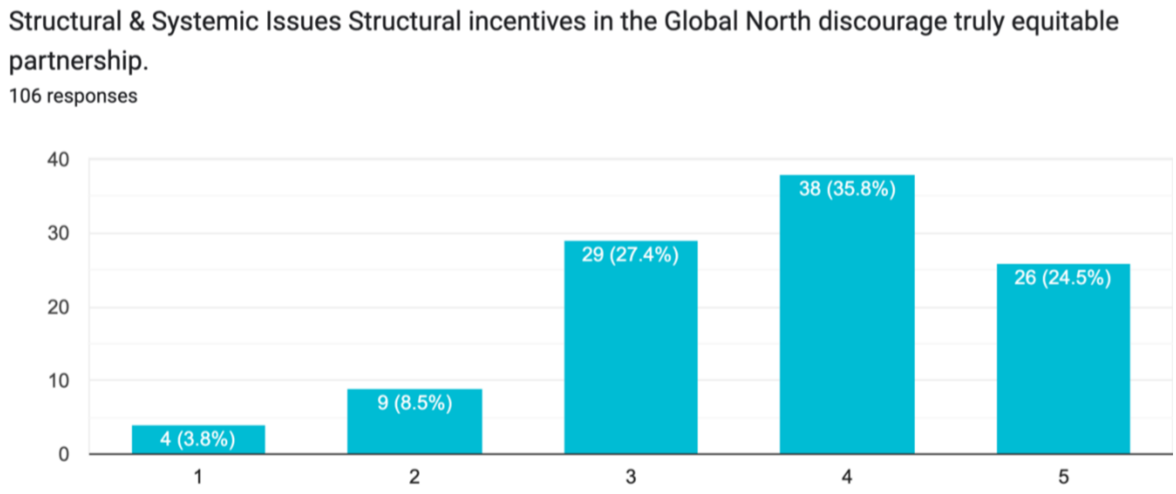

Q14. Being based in the Global South restricts scientific career opportunities

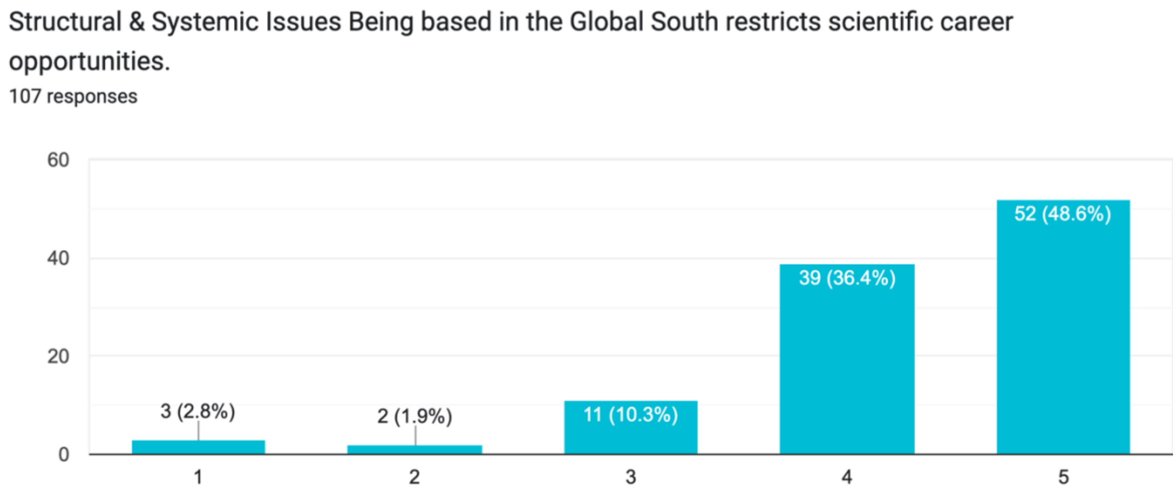

Q15. Researchers from the Global South face significant mobility and visa barriers

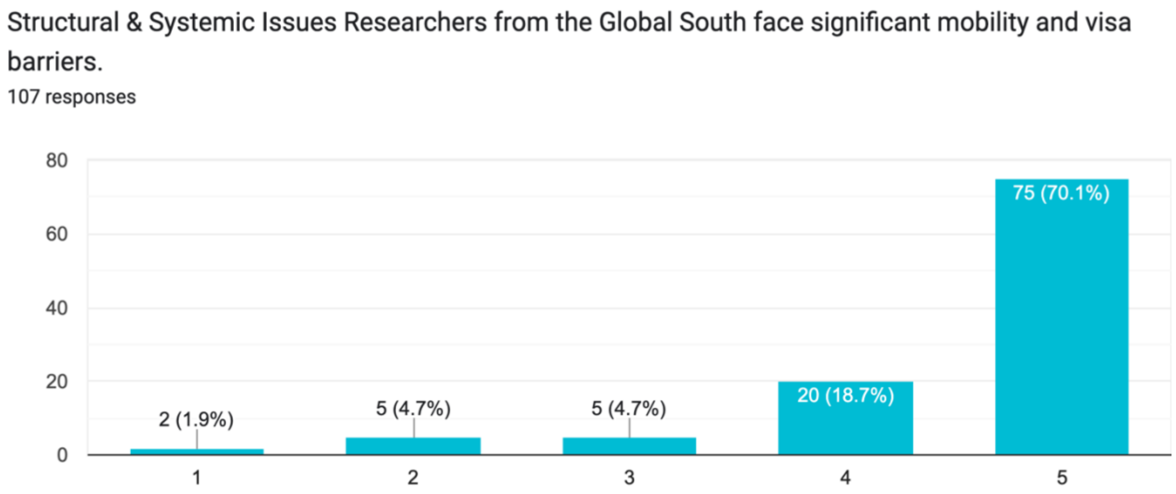

Q16. Diaspora scientists often act as more effective bridges for equity than institution-to-institution agreements

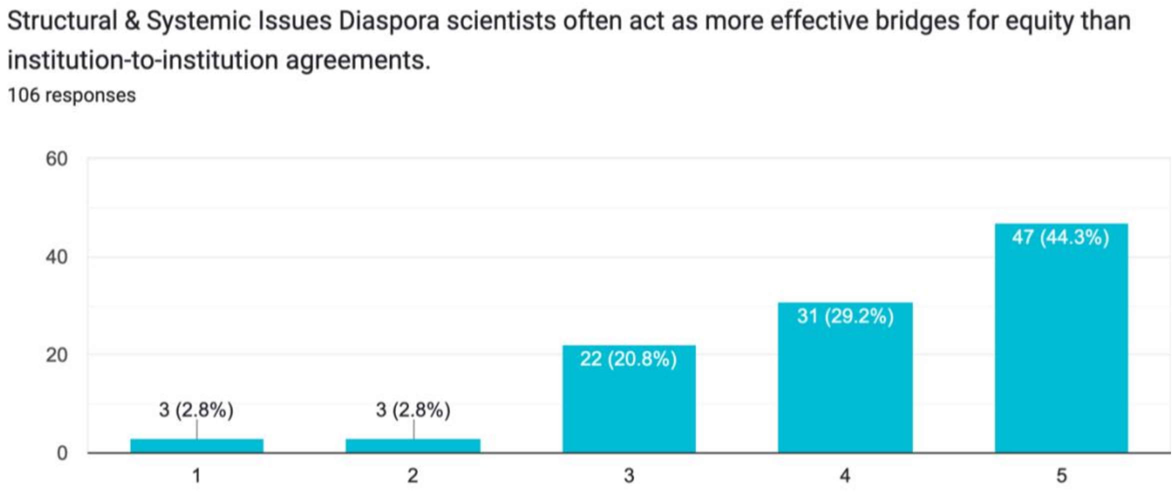

Lived Experiences (Q17 – Q20)

Q17. Some collaborations feel one-sided: data/resources flow South → North, while credit stays North.

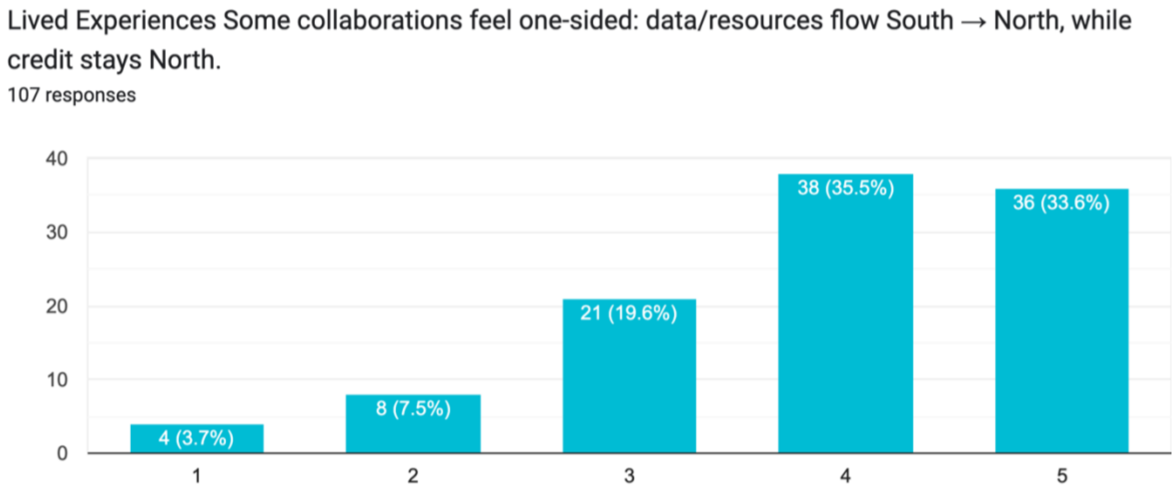

Q18. I have been assigned basic or repetitive tasks instead of meaningful scientific responsibilities

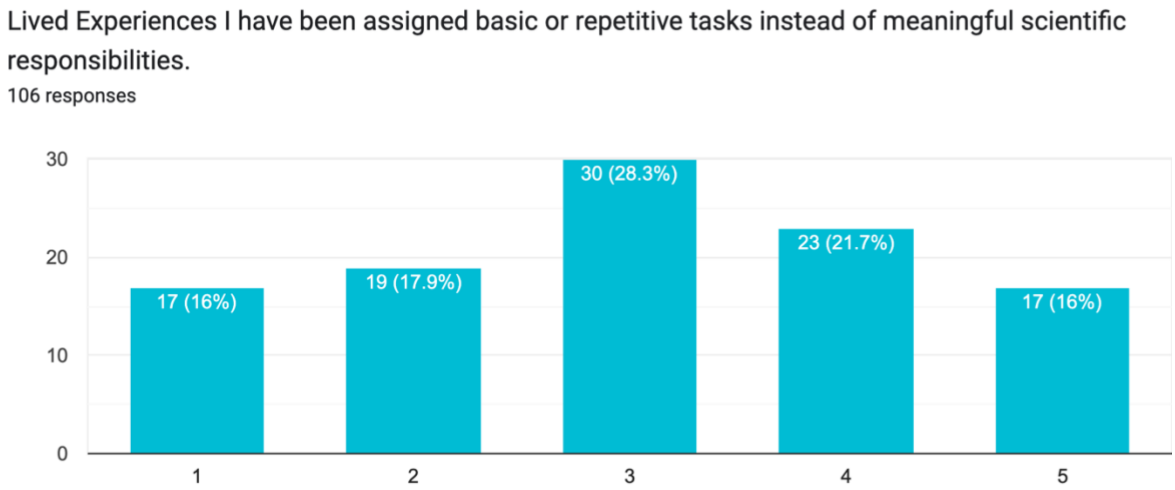

Q19. I have been denied authorship or leadership opportunities that I felt I deserved.

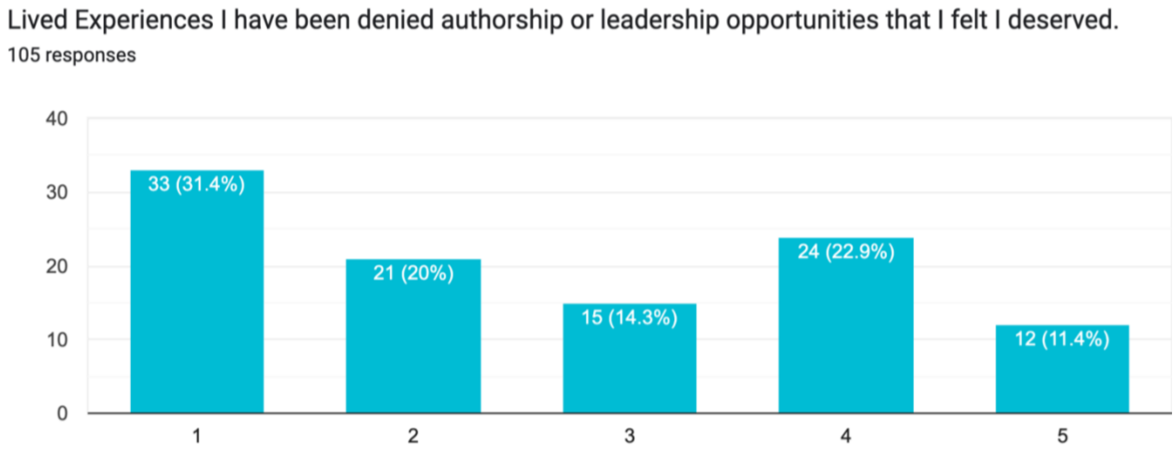

Q20. I have experienced or witnessed subtle discriminatory behavior in global research settings

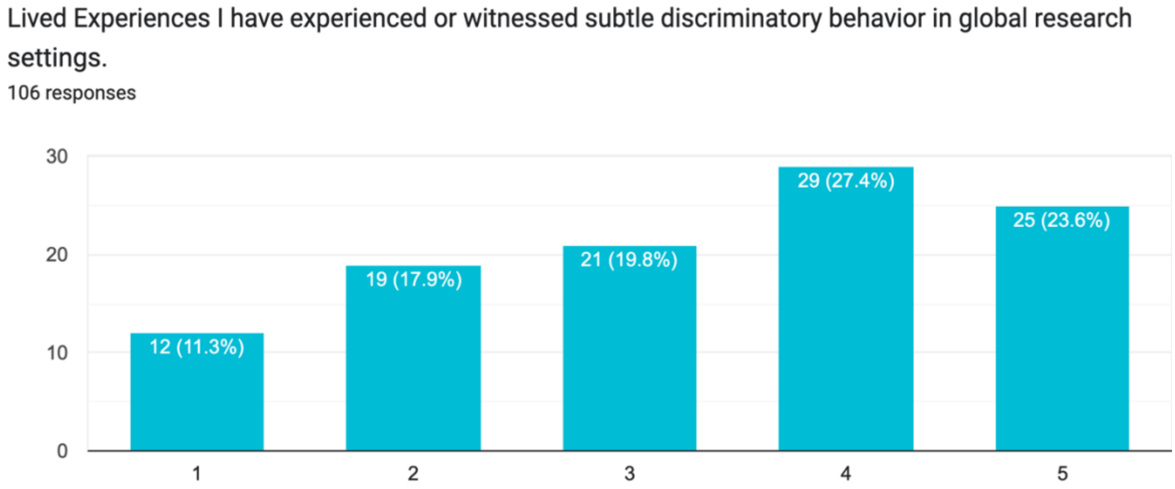

Section 3 – Decision-Making, Ownership, and the Future (Q21- Q26)

Multiple Choice answers

Q21. Who typically holds the real power in Global North–South collaborations?

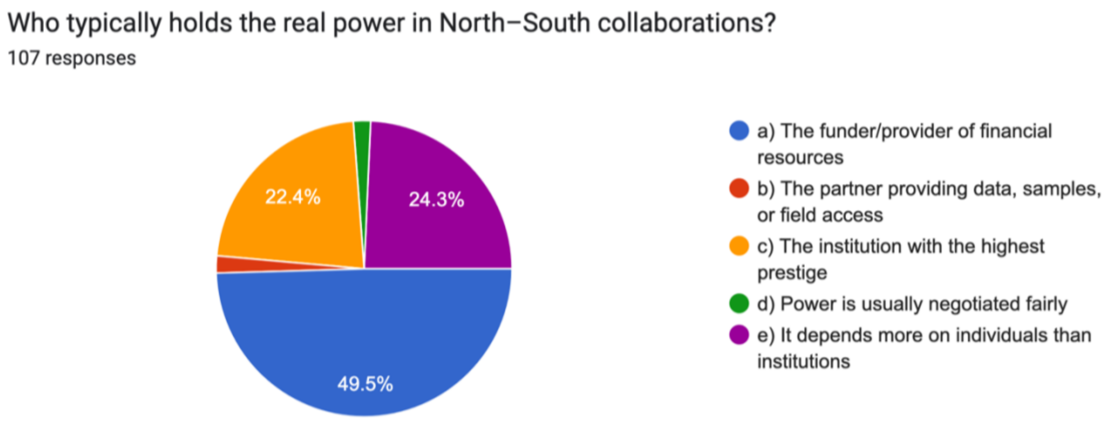

Q 22. The concept of "decolonizing science" is

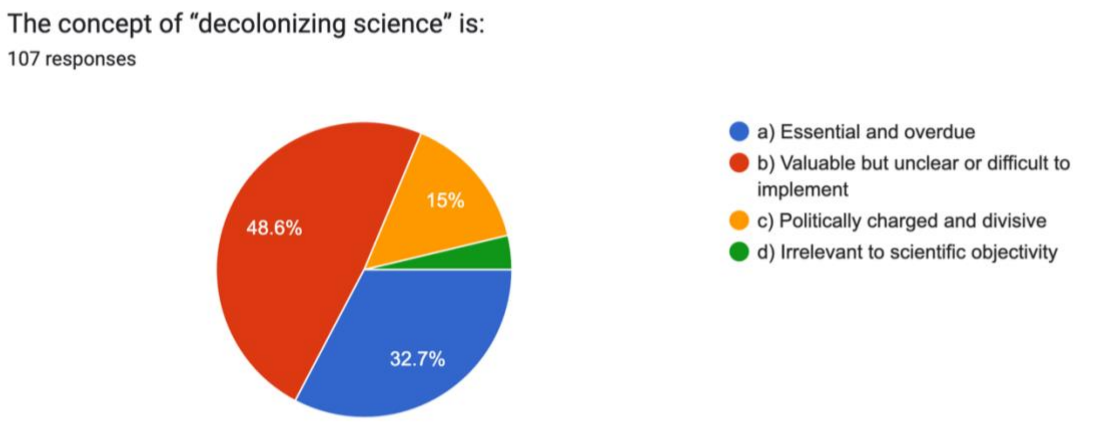

Q 23. Authorship in high-impact papers should primarily reflect:

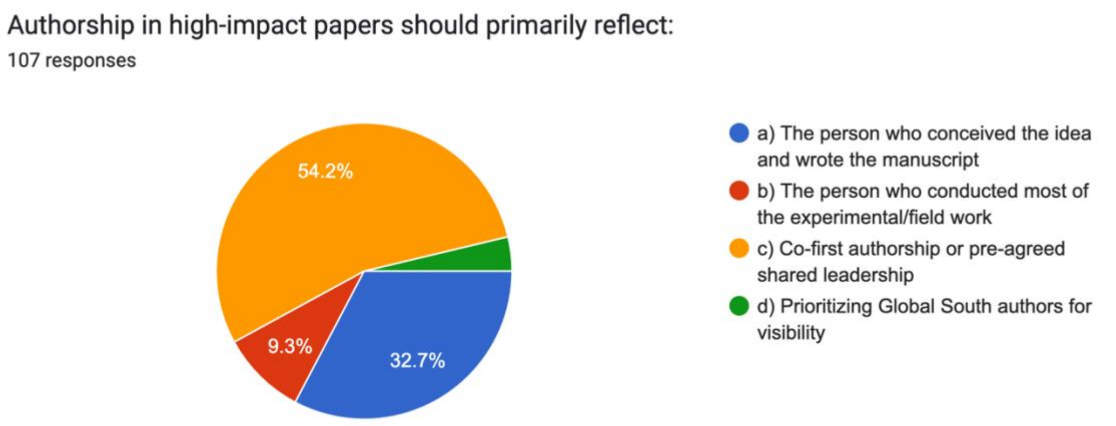

Q 24. The biggest barrier to equitable North–South collaboration is

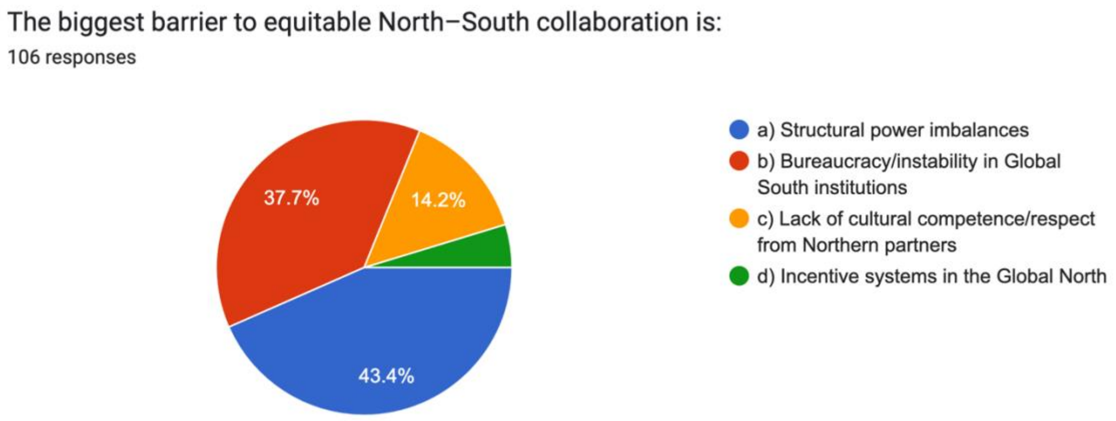

Q 25. How effective are current international funding models (EU, NIH, Wellcome, NSF)?

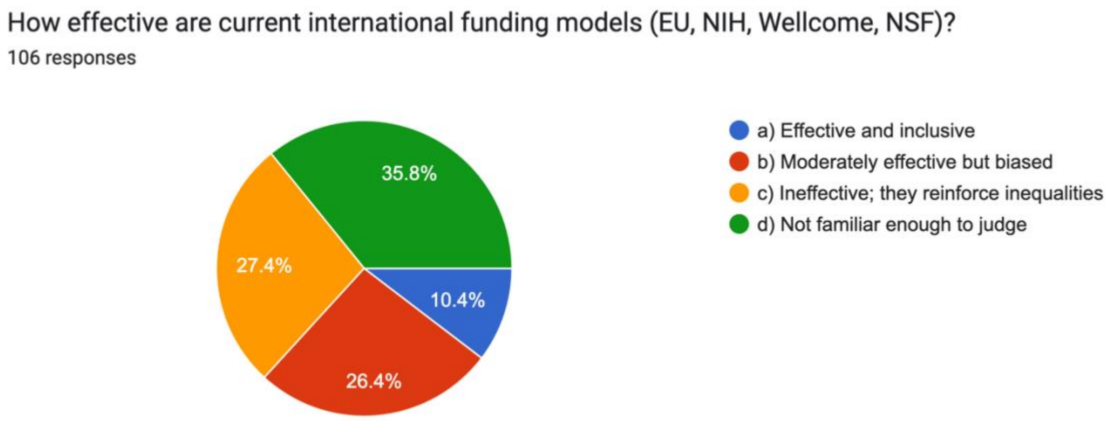

Q 26. The most promising future model for global collaboration:

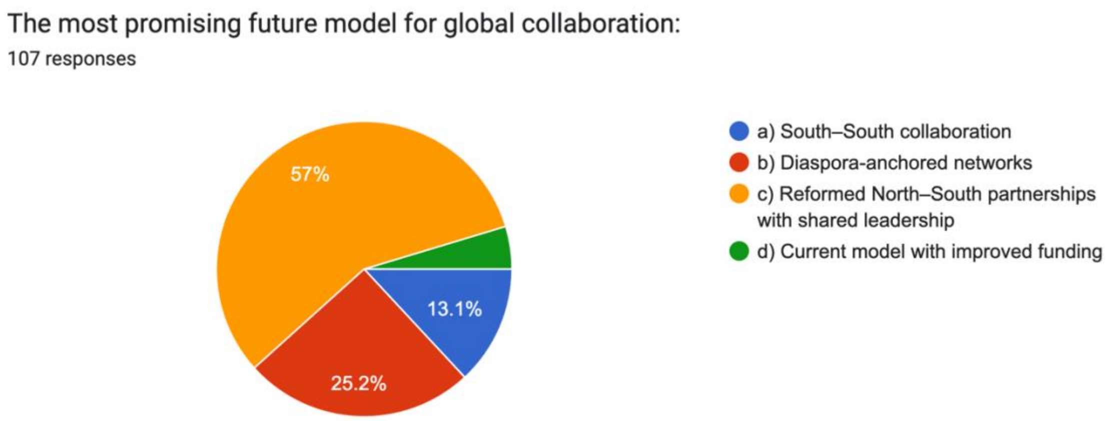

Section 4 – Open Questions (Q27-Q31)

Q27 Describe one experience—positive or negative—that best illustrates your view of North–South collaboration. What was the key lesson?

Woman	During a previous international project on low budget water monitoring, samples collected in a South American country were shipped to a European laboratory for advanced analytical analysis. Local researchers contributed heavily to fieldwork, contextual knowledge, and logistical coordination. However, once the samples arrived in Europe, most scientific decisions like data processing strategies, statistical analysis, interpretation of results, and partially manuscript writing, were made mostly exclusively by the European partners. The final publication listed local scientists traditionally as middle authors, despite their essential contributions. This experience showed me that access to infrastructure and funding (not always but pretty often) determines scientific power.
Man	I once participated in a joint North–South research project in which I provided the legal framework and regulatory analysis for a scientific paper. While the work was incorporated into the final publication, proper academic credit was not granted. This experience highlighted how imbalances in recognition can persist even within formal collaborations. The key lesson was the importance of clearly agreed authorship and credit from the outset.
Man	A key to my positive experience is living in a region near a very important National Park, especially for foreign researchers. This attracts many researchers who require local assistants. Likewise, the researchers' willingness and respect in guiding and helping local assistants, as in my case, has been invaluable.
Man	A positive example was the creation of a collaborative network across several institutions, which provided robust infrastructure and made it easier to connect with researchers. On the negative side, the bureaucracy in my home country posed significant challenges in securing funding for travel.
Man	A positive experience illustrating the value of North–South collaboration was the opportunity to pursue my doctoral studies in Europe (Global North), thanks to scholarship programs and bilateral agreements established with my country of origin (Global South).
Man	All of my at least 10years collaborations between north and south had been great. Non bad experiences. Main countries are Spain, Belgium, USA, Turkie.
Woman	an effective North-South collaboration shares benefits, builds local scientific infrastructure and expertise, and respects the challenges and knowledge of Southern partners, which leads to sustainable and impactful scientific progress. I have seen this with the Indiana University and USFQ agreement of student interchange and research support.
Woman	As a holder of a development-oriented scholarship, my overall experience has been positive. I have felt well supported by my supervisors, and the funding has enabled me to pursue both a master’s and a doctoral degree that would otherwise not have been possible. For this, I am genuinely grateful.<br><br>Nevertheless, I believe that some structural challenges were not fully considered in the design of the collaboration, despite their direct impact on the success and well-being of doctoral researchers under this scheme. I travel frequently to my main institution, and I recognize that my funding model is based on an allowance rather than a salary. While I understand the rationale behind this structure, in practice it often makes me feel more like inexpensive research labor than a fully integrated member of the academic community. This disparity becomes particularly visible when compared with native PhD students with whom I share an office and daily work environment. They benefit from stable salaries, paid holidays, and do not face constraints such as having to complete sample processing before a visa expires, searching for accommodation every few months, or repeatedly applying for visas—yet we are expected to meet the same deadlines for thesis defense.I think these conditions can generate tension and impose an additional psychological burden on researchers in similar positions. I do understand that people that see these benefits just prefer to stay in the Global North and foget about the South.<br><br>Thus, while this funding scheme is highly valuable in enabling access to academic degrees and research opportunities, it does not sufficiently take into account the social, psychological, and financial challenges faced by researchers from the Global South who must navigate multiple systems and expectations. I strongly believe that development-oriented funding programs would benefit from a more person-centered approach—one that considers not only academic output, but also dignity, inclusion, and long-term professional equity.

Man	As the world expert, I was asked to collaborate in a paper in which I was clearly asked because I am the expert, but was placed as last of 5 authors (the others young inexperienced students but anxious), despite the fact I knew the taxa and had the most experience with the taxa and knew their relationships and they did not. I felt a bit "used" but wanted to encourage their interests and collecting activities.
Man	At a conference, I shared an idea for a research project. Years later, some U.S. researchers who attended that conference invited me to participate in fieldwork for the same project. The key lesson is to be cautious when sharing ideas freely.
Man	At the beginning of my PhD people underestimate my capacities because my origin
Woman	At the end, I was a logistic and permit provider. My lesson was not to waist more time with those "collaborators" and focus my energy in meaningful relationships
Woman	Choose your collaboration carefully and make sure that they value your ideas from day 1.
Woman	Collaborate on a study between UK, Chile and Argentina. All good researchers but unfortunately those in Chile and Argentina had significantly less time to dedicate to the project
Woman	Colllaboration is always pretended wheit comesto realitynoone isat home
Woman	Democratising microscopy in Latin America workshop funding by the company of biology 95% of speakers were people doing science in Latin America countries doing high tech science
Man	During my master's degree, I was offered the publication of my work as part of the requirements for obtaining my postgraduate degree. This ultimately did not happen, and the results of my work remain unpublished.
Woman	During my master's research on quality of working conditions, I observed how knowledge from the field—particularly from local actors—was essential to understanding realities on the ground, yet often remained under-valued in formal academic frameworks. Even without being part of an international publication, it was clear that decision-making power and theoretical framing tended to remain concentrated in more institutionally recognized spaces.<br><br>Key lesson: meaningful collaboration requires recognizing experiential and contextual knowledge as full scientific contributions, not just as supporting data.
Woman	Equal partipation among researchers Project BIOGEEC. - Have an Ecuadorian and German coordinators of the project improved our perspective of the project and work as equals was funddamental .
Woman	Equal partnership between North and South requires local partners and cultural knowledge. I.e. research about Chile done by Chilean diaspora rather using international funding rather than being led by international researchers with little knowledge.
Woman	Even if North collaborators think about South collaborators, they have to be aware of the privileges they have. For example, native English-speakers are usually not aware of their privilege in reading articles in their native language, or about their privilege that a lot of scales and procedures are developed initially based on their culture or language, which facilitates their research time.
Woman	even strong initiatives from the South are often overlooked unless validated by the North. True collaboration requires negotiating leadership and recognition from the beginning to avoid reproducing these asymmetries.
Man	Fish model provided by south country and publication by a north country with all people involved cited in authorship
Woman	for a paper I was invited to co-author. I was asked to collaborate with materials from the southern hemisphere that are basal and very complex to find,
Woman	For centuries, collaborations to describe the flora of southern countries have been led and monopolized by researchers and institutions from northern countries.
Woman	Forest networks, they share the autorship
Man	From a scientific perspective, there is a dominance of the Global North over the Global South, evident in terms of available funding, which impacts the potential for scientific advancement. Furthermore, high-impact journals often require more information when discussing topics related to the Global South. This situation is frequently problematic because less research (resulting from a lack of funding and government resources) creates a gap that widens the divide between hemispheres.To figth this, one alternative is to keep researchers outside their countries of origin in the Southern Hemisphere, supporting the flow of funds to the Southern Hemisphere. This can be achieved through scholarships, research funding from the Northern Hemisphere to the Southern Hemisphere, collaborations between research centers, and other initiatives. Similarly, governments should seek ways for their researchers to pursue careers abroad and contribute to the global economy through these types of solutions.

Man	Generally, in the projects I have participated in, the proposals are already made for the collaborators, and ours is only a secondary participation. That is, conceptually, the project is conceived by them, the methodological design is theirs, and we only participate in the collection of field data and in the articles we will be co-authors, but we will never lead the research.
Woman	Getting grants positive granted is a problem most of the time th poolitical persons somehow collaborate and you get a little of the pie. This now for several an structural patron
Woman	Global North researchers believe that South global researchers are less competente and prepared
Man	Global north researchers come to the South with no permits, hire students or local enthusiasts, do the research and leave with all data and no benefit for students, researchers or institutions in the South.
Woman	Have not yet experienced something
Woman	He perdido mayor oportunidades y recursos al norte que en sur américa
Man	he results are taken abroad and serve other countries
Woman	High cost of the Publications
Man	I collaborated for over 4 years with a team in Cape Town, South Africa. The scientific experience has been fantastic, they are incredibly talented, but their burocracy and admin issues are way more complex that those in my insitution in the UK. We have stopped working together this year cause their capacity to run experiments has been discontinued due to financial decisions.
Woman	I don't have enough experience (a am a "northern" and moved to another "northern" country)
Woman	I got grant to work in London for a period, a man look for me and work together, even not specialist o the group. When I returned home, he starts working in the same group, 3 years after he started publishin my results, initially inviting me and my colleagues, after only with researchers from England
Man	I had the opportunity to get an internship in a botanical garden of USA
Woman	I have written a research project that is at the moment compiting for a Welcome funding. I have found a Chilean institution as collaborator centre, I am acting as a bridge between that center and a UK university. The university gave a lot of support an extra funding to apply to welcome and move on my project, the collaborator centre has given me access to relevant archived. The results of the investigation will be in benefit of the Chilean<br>community that was an exigence from Welcome, and the UK university agreed with it.
Man	I havent had any exoerience
Man	I offered a german colleague to be part of a project and enrolled him as a supervisor in one of our graduate programs 14 years ago. He has so far supervised half a dozen students and the students have always been priority and first authors of all articles.
Man	I participated in a project between institutions form North and South that involved professional development given by the North institution. Collaboration was difficult because of red tape in South institution, and costs being prohibitive for the South.
Woman	I provide data but I am not a coauthor
Woman	I received support from the EU at a time when Colombian institutions left me without support, in a situation that I had chosen based on professional ethics
Woman	I was invited By a american researcher to participate in a field trip to collect fishes in Ecuador, only to be asked to fill tubes with alcohol. Then I found out he needed a woman for DEI requirements.
Woman	I work as a museum coordinator. Most external collaborators still view us as an (overqualified) operations and processing base camp without realizing all the soft skills we provide to allow their science and do not offer usually equitable collaborations
Woman	I’ve had several North–South collaborations that worked smoothly, but one experience stands out because of how well it worked. I was part of a project where the Global North team arrived with expertise, but without the typical “ready-made agenda.” Instead of prescribing solutions, they asked local teams what the priorities were, what was feasible, and what would actually benefit the population. That changed everything.<br><br>The key lesson for me was that collaboration succeeds when both sides step in as equals. Trust grows when the North respects local knowledge, and when the South feels safe to lead and question without being overshadowed.

Woman	If you investigate about South you have more responsible point of view.
Woman	In a scientific conference a British researcher helped a Namibian collaborator who couldn't attend
Woman	In many circumstances, basic rights need to be reinforced. Such as co-authorship for those collecting the data, strong data, sharing agreements to protect the ones collecting the data and acknowledgment of support from local institutions.
Woman	Is very difficult but not imposible. People from North are a bit redundant to work with people from the south. I experience collaboration between Germans and Brazilians PI and is a fight of power. At the end, the relationship broken definitely with them not talking anymore and no more collaboration.
Man	It is hard to explain to colleagues from the Global North what the realities of the Global South are, in terms of research and academic structures. Everyone seems to think that the world works "ideally," and that the protocols and conventions of the Global North are universally appliable, but this is not the case.
Woman	It is more of the North holding the purse and getting the south to align. The south should learn to step forward and prove its competency.
Man	Know the collaborators before starting any agreement. I think it's super important to establish and agree with the roles that the partners set.
Woman	lack of shared leadership
Woman	Los eventos que se realizan en el norte discriminan a los del dur limitando sus visas
Woman	My best expirience was with my advisor at the UF ! Finding the wright one was key for a starting career! Is the only way for not being crushed by the system from the beginning!
Man	Na
Man	Negative, authoritarianism
Woman	Negative: Survey based research where Global South partners are mostly involved only as gathers of participants, but then almost not included in writing and discussing the paper.<br>Positive: Studying and researching for about a decade in Europe, then returning to Ecuador with new and updated perspectives on the global tendencies to research for solutions to local impacts in Ecuador, having a rich portfolio, knowledge and networking skills and connections.
Man	no tengo conocimiento
Man	North researchers are not willing to be open to ideas provided from South researchers, usually tendening to focus on north concerns or hypothesis
Woman	Northern institutions still see south universities as: " parners to get data" but completely understimate people intelligence. (Maybe bacause of bad experiences?)
Woman	Northern partner, specifically Norwegians, whith questionable scientific rigor and taking credits for the work conceptualized and executed in the South. Also being unfair in economic resource distribution, unilaterally benefiting themselves with Lack of transparency in project economic management
Woman	On a field trip in the jungle with Europeans, we shared the same activities, and if they had the most knowledge, they would explain things. But when I went out with a Japanese group, they were the ones who led, limited and defined the observations and joint work.
Woman	One experience that shaped my view of North–South collaboration happened during a joint health project between a European team and a local clinic network in Central America. At first, the Northern partners arrived with a fixed plan that ignored local knowledge, and the project stalled. Everything changed when the local team challenged the timeline and was invited to redesign the approach. Once their expertise guided the project, the work became more efficient and culturally grounded.<br><br>Key lesson: True North–South collaboration requires sharing power and valuing local knowledge from the start; otherwise, even good intentions fall short.
Man	Our participation is based more on contact with common scientific groups than on the obligation to include collaborators in the global south.
Man	Over the years, a marked bias and a profound gap between the Global North and Global South have become evident in terms of scientific research and publication, in many cases, those of us working from the Global South must invest five or six times more effort, sacrifice, and discipline to achieve comparable outcomes, moreover, factor unrelated to scientific quality such as institutional origin or, at times, even our surnames may influence whether a manuscript is accepted or rejected by high-impact journals, a situation that researchers or students belonging exclusively to institutions in the Global North rarely face

Man	People demanding DNA resources, with the typical "send me all that you have", instead offering coauthorship (Negative)<br>People offering training in new techniques (genomics) including the trip and expenses, all intended to be included in a project and then a paper (positive)
Woman	positive: with North funding (EU), engaging researchers with origin in the South (diaspora) collaborating with researchers in the South with shared learning, and effective results
Man	Revisión de un género, desde el trabajo de campo hasta la herborizacion,datos bibliográficos,claves no reconocidas ni en agradecimientos ni publicación
Man	Shared allocation of responsabilities, where the upstarting spouth researcher acquares experience and knowledge from the north "experts"
Man	Since I joined an international association my research outcomes improved a lot.
Man	The creation of opportunities for southern university students to interact with northern university faculty.
Man	The process in the diplomatic relations with others . The key lesson is “Give and take “
Man	The space to express ideas has to be similar for all participants
Woman	The universities in Global south have strong potential but extensively lack resources. More resources would greatly open up possibilities and attract more mobility
Woman	There are excellent programs that prioritize or allocate resources to people from the Global South to participate in workshops and conferences featuring leading scientists explaining or teaching about cutting-edge technologies. Some of these opportunities don't reach scientists in the Global South directly, but rather remain within a very small circle of scientists, thus failing to reach the vast majority of communities.
Man	This is not an academic example but the lesson applies also to academic collaborations. The team that won the international 10 million dollar Rainforest XPrize last year was led by a US biologist who started his biology career in Ecuador, and who maintained close personal relationships with his many Ecuadorian partners, particularly his indigenous guides. His team included many of these indigenous experts. Upon winning, he helped his indigenous partners form a biodiversity assessment business and they are now doing rapid assessment work in Ecuador and other Amazonian countries. The successful collaboration was due to the genuine personal friendships and respect built over many years. I believe this is one of the keys to good collaborations. They cannot be imposed by institutional agreements, they have to be earned by the personal effort of all participants.
Woman	Unfortunately the monetary collaboration takes up over the knowledge. Typically North countries have this money and the scientists that come from South are not visible when a paper is published because the principal author comes from the North since this person puts the money
Woman	Usually, at least in my country, Ecuador, it is enough for someone to be a foreign professional—even if they come from Peru or Colombia, countries with which we share borders—for people to listen to them and almost praise them. This does not happen when you have the same experience and knowledge as an Ecuadorian yourself; you are not invited, and even less appreciated. Imagine what it’s like when the speaker comes from the First World.
Man	Varios proyectos con fondos británicos para desarrollo científico pesquero; con fondos belgas, para desarrollo acuícola, con fondos de USA para investigación climática. Todos proyectos satisfactorios que aportaron con investigación científica y formación de talento humano.
Man	We associeted complentary expertises in pluridisciplinary field to publish.
Man	While bias in the evaluation of project proposals also occurs in the Global North, it is likely more pronounced in the Global South. Institutions in the South should adopt fairer selection processes to identify and highlight the most promising proposals. This would probably incentivize North-South collaborations.
Man	
Woman	
Man	
Man	
Woman	
Woman	

Woman
Woman
Woman
Man
Woman
Woman
Man
Man
Woman
Woman
Woman
Woman
Woman
Man
Woman
Woman

Q28 Do you see “brain drain” as primarily a loss, or does “brain circulation” create overlooked opportunities? Explain.

Woman	Brain drain is mostly a loss but it has the opportunity to become brain circulation if the right conditions are given. When countries make it easy, rewarding, and practical for scientists abroad to stay involved. If they receive support and see benefits on both sides, researchers stop being “lost” and instead become bridges.
Man	Brain circulation is not optimal for the global south; however it does contribute to science in general.
Man	Yes, I consider a loss.
Man	I see it more as brain circulation than brain drain. Today it is far easier to establish partnerships and to visit leading research centres, which opens up many opportunities. At times, conditions abroad can be particularly attractive, especially when specific analyses or resources are required. However, returning to one’s country of origin — particularly if there is a stable, permanent position available — offsets what is often described as “brain drain.”
Man	The crucial thing is not to prevent professionals from leaving, but rather to establish mechanisms to capitalize on the knowledge and networks they gain abroad, putting them at the service of national progress.
Man	I don’t have enough background relates with Brian drain tópic
Woman	The key insight is that brain circulation transforms brain drain into a potential brain gain. Returning migrants or diaspora members act as intermediaries, facilitating scientific and technical cooperation, foreign investment, and knowledge transfer between countries. This promotes sustainable growth in the origin countries while enriching the global scientific community. However this depend on the country that recieves back the scientist, usually the returning academic is miss treated, opened humillated since this person is no loguer in the local network, you miss a lot of opportinities and takes a lot of time to beging again. The local institutions do not always have the culture to recieve the returning migrants and help them to faclite cooperation.

Woman	I do see brain drain as a loss, but I would not blame the individuals. I understand why the choose certain path. I do believe those people some of them will open doors for newcomers. Nevertheles, I think the talent will end up working in areas needed for the development of rich countries leaving behind the poor ones. It is a pitty.
Man	Brain drain is a loss, but understandable due to structural capacities and opportunities and politics.
Man	“Brain drain” is often framed as a loss because it involves the departure of highly trained individuals whose education was frequently supported by their home countries. This can weaken local institutions, reduce research capacity, and slow social and economic development, especially in countries with fewer resources.<br><br>However, viewing the phenomenon through the lens of “brain circulation” reveals important and often overlooked opportunities. When skilled professionals move abroad, they can build international networks, gain access to advanced infrastructure, funding, and new ways of thinking, and later transfer this knowledge back to their countries of origin. This transfer does not always require permanent return; it can occur through collaborations, joint publications, technology transfer, mentorship, and policy advising.<br><br>Therefore, the impact depends largely on structural conditions. In contexts where institutions maintain strong ties with their diaspora and create incentives for collaboration, mobility can become a catalyst for innovation and capacity building rather than a permanent loss. In this sense, “brain drain” and “brain circulation” are not mutually exclusive concepts: without supportive policies, mobility results in loss; with them, it can generate long-term collective benefits.
Man	Brain drain is primarily a loss for the Global South if researchers who emigrate do not contribute back to their countries of origin. However, if these researchers engage in "brain circulation" by sharing knowledge, investing, or returning, it can create significant opportunities through knowledge transfer, remittances, and enhanced global networks.
Woman	Brain circulation sounds better to create opportunities
Woman	Brain circulation can be effective when there are resources (human and infrastructure) in the South
Woman	I believe that the opportunities that researchers can get are vital to promote collaborative research. If researchers don’t move between centres, it is difficult for big centres to see small ones and for the latter to achieve good impact and development.
Woman	Brain drain comeswiyh no. opportunity tomove forward
Woman	It helps to create bridges from the north and the south. So this person who has move it helps to create this new bond
Man	I tend to think that "brain circulation" creates opportunities that, for various reasons, could not be developed in the countries of origin. However, this further exacerbates the imbalance with the North, as a consequence of the "brain drain," primarily from the South.
Woman	From my perspective, brain drain represents a loss when mobility leads to a disconnection from local realities and institutions. However, brain circulation can create important opportunities when individuals maintain strong professional, intellectual, or mentoring links with their country or region of origin.<br><br>The issue is less about individual mobility than about the lack of structured mechanisms that allow knowledge, skills, and resources to circulate back in a sustainable way.
Woman	At least in Ecuador, brain drain is more important , becuase people loss connection with their native country and that is a problem
Woman	I see "brain gain" only. In a global world, science does not have geographical or political limits countries have.
Woman	Both. As part of the psychology field, I've seen that there is a lack of international diversity in this field, so the brain circulation provides a new vision to the field. However, there is still a brain drain, and this difficults to see differences on research across the world (e.g., cross-cultural studies).
Woman	For me, brain drain is primarily a loss. Even though brain circulation can generate visibility and networks, these rarely translate into concrete benefits for the country. In many cases, the knowledge and connections gained abroad do not return in ways that strengthen local institutions or address structural inequalities. So while circulation has potential, the reality is that the South often loses more than it gains when its trained professionals leave without the conditions to contribute meaningfully back home.
Man	Yes

Woman	Brain drain is something very dangerous for a developing country like Argentina. It shouldn't happen massively like it's happening right now.
Woman	Both things happen; in some cases, when researchers are leaders of research groups in their regions, a large gap is created, but on the other hand, there are no mechanisms in place or very few facilities to ensure that this acquired knowledge can be shared in countries of the south.
Woman	“brain circulation” create overlooked opportunities
Man	When you initiate external investment processes, it ceases to be a drain and becomes a direct contribution. From a country's perspective, you can generate significantly more revenue if you have a validated and coordinated investment plan. The opportunities to improve science in Latin America today lie abroad. Until there is genuine interest in developing the region, very little can be achieved with investments below 4% of annual GDP.
Man	Both can be valid. Many “brain drain” individuals do not collaborate with countries where there are no budgets for science or where institutions do not allocate resources for research, and they seek other opportunities in other countries. However, “brain circulation” individuals can seek opportunities and form strategic alliances to promote research by combining strengths that are not necessarily economic.
Woman	You get pay very bad and orth come give you a little money just enough to do the basic research took your idea and they are gone . And in that way we facilitaate brain-drain
Woman	Brain drain is a loss of big talents for GS countries since many of this talents for sure would like to stay at their home countries and are force to work abroad. Even if the people would like to come back, the opportunities in the home country are not adequate for the level of education/preparation they have. Which make them to seek for opportunities they find in the global north. Brain circulation with diaspora programs would be a good way to collaborate from abroad, if the programs regulate work exploration and bad practices in collaboration with researches of the diaspora.
Man	In my opinion most of the diaspora researchers behave the same as researchers coming from the north.
Woman	Brain drain is a primarily loss
Woman	Crea oportunidades porque muchas veces se valora más el trabajo y la calidad de información en otros sitios diferentes al del de investigación
Man	f course, due to the lack of incentives and assessment of capabilities, researchers go abroad and serve other countries
Woman	Opportunities appear if the circunstances are good
Man	Brain drain doesn't make sense in a globalised world, and it is also disrespectful to the talent that stays. The real issue is creating the opportunities for real collaboration. Maybe more than just opportunities, is real incentives (like making them part of the payment for scholarships, where you have to collaborate and publish with double affiliation and with partners in the country of origin)
Woman	In my experience most of researches who moved to the North stay there, only few of them collaborate with home country
Woman	Brain drain is a loss to the original country. Some never go back home, and sometimes not returning the knowledge
Man	I don't know
Woman	Chile doesn't count with enough money to invest in research, so to circulate allow researchers to find more opportunities to fund their research. Also it put us in contact with a global world of knowledge to be included in.
Man	Is a loss
Man	Brain circulation is good to refresh science. And what may look like brain drain can turn out to be rather positive when this researcher starts accepting graduate students from the global south
Man	Brain drain is kind of a loss because a stronger structure for growth is better exploited in the north.
Woman	Colleages that go abroad want to work by themselves rather than their colleagues from their country
Woman	yes, “brain drain” as primarily a loss
Woman	I could not tell.

Woman	It is a primary loss, also because if Global South colleagues relocate to the global north they often do not reflect on their privilege and replicate inequal models and practices
Woman	I see both sides, but honestly, the term “brain drain” is too narrow to describe what’s happening today. Yes, countries lose talent, and that hurts. I lived that, leaving Brazil, then Ecuador, then the U.SS, and I know what it means for the local system. But we underestimate brain circulation: people who leave, learn, and later build bridges back home. When institutions support that flow—mentorship, data-sharing, joint grants, visiting positions—the Global South gains access to networks and tools that would otherwise take decades to develop. The problem is not people leaving; the problem is when systems make it nearly impossible for them to reconnect.
Woman	I think it is a loss for countries.
Woman	Brain circulation are overlooked opportunity, having nationals of the G.South in the North con promote collaboration, student exchange, finding cycling
Woman	Currently brain drain is still a loss, but I am optimistic that we can transform that to brain circulation. Much of the diaspora is so overwhelmed with their academic work load that it’s difficult for them to include work in the global south.
Woman	I don’t find it is a loss. The problem for me is when you want to come back to your country and you don’t have the opportunity or all the doors get closed.
Man	I think brain drain is a problem, but I also think that brain circulation is a huge opportunity. In a world characterized by inequality, though, brain circulation turns to brain drain quite automatically.
Woman	Brain circulation is advantageous and tends to reduce the ultimate affect of brain drain
Man	Not sure how to answer
Woman	It is a loss, because it means losing the opportunity to strengthen science and technology for the benefit of the country's development.
Woman	Yo lo veo como una pérdida,pues puede resolver en su país problemas en su sociedad
Woman	
Man	Loss
Man	a loss irreversible
Woman	It is gain on connection and knowledge as much as it is actively handled and recognized within this frame, even a scientist abroad is an anchor and asset for origin country.
Man	en mi Pais(Ecuador)es malo
Man	Brain drain is a loss. Many of the scientists from global south are not willing to work with colleagues from south
Woman	Most of the scientist in the global south want to go to the north and not necessary collaborate with the south! so yes brain drain is a problem.
Woman	Brain Circulation is a must to connect scientists to prioritize scientific development with resource optimization, over geographical location and ego
Woman	I think it's great that professionals go abroad to improve their skills; but it's important to encourage their return since their contribution and scientific advancement are significant.
Woman	I see brain drain as a loss only when people leave without meaningful ways to stay connected. But when mobility allows for ongoing collaboration, knowledge exchange, and investment back home, it becomes true “brain circulation.” In that case, movement of talent creates opportunities rather than depletion.
Man	It is a loss due to lack of opportunities, corruption, and lack of funds.
Man	There is clear evidence of the ongoing brain drain of the best researchers from the Global South, who, upon migrating to the Global North, undoubtedly find greater opportunities and better conditions for professional development, however, many of us who love our countries and wish for them to be visible on the global scientific stage choose to remain, even while fully aware of the limited or nonexistent resources available for research. This is also part of our commitment, to be resilient and to pass that strength on to future generations, it is part of our identity as Latin Americans to never give up, to adapt to any environment, to fight, and to move forward even with the few resources we have.
Man	Being a expat means that your native country and universities are losing oportunities

Woman	brain drain is still relevant to study causes for emigration, but should not be the dominant paradigm any longer, brain circulation is the present and the future
Man	La fuga originó vacíos de especialistas en taxones específicos,y no hubo preparación de relevos en esas áreas,además la situación país hizo que migraran y muchos de dediquen a otras actividades u oficios
Man	primarily a loss, because those researchers, although may collaborate with their country of origin partners remain mostly in the north sphere of influence
Man	Brain circulation is a potential opportunity.
Man	I think both of them occur in a certain matter.
Man	N/A
Man	Yes brain drain is mainly a loss
Woman	Loss: for someone leaving their country due to limited financial possibilities, it is a real loss for the country.
Woman	The brain drain is definitely a problem facing the Global South. Academia and research in countries of the Global South are largely new, meaning there is a lack of understanding of how to manage them to create opportunities. Furthermore, there are few university programs that support scientists abroad to continue their research or collaborate with scientists in the Global South. Adding to this is the heavy workload of scientists worldwide. The administrative, mentoring, and research responsibilities are so numerous that they leave very little time for building new partnerships.
Man	Both. Ecuadorians in foreign countries are badly missed in their home countries, but they forge research connections between their home country and their adapted country.
Woman	It’s a loss mainly in our countries. People who had the capacity had to move to North countries to have the opportunity to make new researches since in our countries there is no investment in this programs. At the end north countries benefit from this brain drain and it’s a loss for us.
Woman	For me, it is primarily a loss, and in many ways. Economic, because the country invested in that person, especially if the professional studied at a public university. Social, because they do not give back to their community what they have learned. Emotional, because they move away from what is theirs, from their loved ones. Moral, because they often convince themselves that leaving the country was ‘the best’ choice instead of staying despite the adversity.
Man	Genera las oportunidades que los científicos de alto perfil no tienen en sus propios países, sumado al tema de seguridad y a la, en general, mala administración de las instituciones, obliga objetivamente a los científicos a emigrar y buscar espacios en otros países donde sus conocimientos puedan ser valorados.
Man	It create Overlooked opportunities, because the diversity of culture provide attractivness to collaborate and differnte point of view for research strategy
Man	The outcome would probably depend on the specific scientist in question. If researchers continue their scientific work abroad, it can be seen as a mutually beneficial arrangement.
Man	
Woman	Brain drain is a loss. We need to create new ways and jobs for the future scientists of the country. When one scientist goes to outside of the country we loss his o her expertise on research, connections and new discoveries.
Man	
Man	
Woman	
Woman	
Woman	
Woman	
Woman	Brain circulation is absolutely an opportunity. It is much easier for members of the diaspora to leverage resources in "richer" countries and use them to do research in the south or to involve colleagues there. With incentives to maintain the contact between scientists and their country of origin, there would not be any brains drained from the

	south, but more like the creation of ambassadors; new pathways to use scientific resources from the north to benefit knowledge about and for the south, as well as south-based scientists through meaningful and equal collaboration.
Man	
Woman	
Woman	
Man	
Man	
Woman	
Woman	
Woman	
Woman	
Woman	
Man	
Woman	Yes
Woman	

Q29 If you could change one rule or norm in global science to make collaborations more equitable, what would it be and why?

Woman	The shared leadership. In many cases, the North decides the topic, controls the funding, sets the agenda, and defines the technical aspects of the project, leaving little room for the South, as key decisions have already been made. If, instead, leadership is shared from the start, power, authorship, and benefits naturally become more balanced.
Man	Global scholarships funded by either n governments paid by debt bonds.
Man	Provide more funding and support to students who are starting out in science
Man	If I could change one rule in global science, it would be to reduce bureaucracy in the exchange of samples and in the processes required to establish partnerships or travel abroad for scientific purposes. Whether it involves sharing equipment or sending and receiving materials for analysis, the current administrative hurdles are unnecessarily frustrating.
Man	In projects utilizing resources from low-income regions, shared leadership and principal authorship must be enforced with local scientists, prioritizing their intellectual role over the logistical one.
Man	Funding
Woman	I would remove the age limits typically applied to defining early, mid, and senior career stages, especially for women scientists. These age expectations do not realistically reflect career trajectories in the Global South, where social, economic, and structural challenges often delay academic advancement. Additionally, I would eliminate the mandatory requirement for scholars educated abroad to return and struggle within systems disconnected from their academic preparation. Instead, funding should support scientific diaspora to work locally or virtually from their home regions, enabling them to contribute without facing institutional barriers alone. This approach fosters sustainable local capacity building and recognizes diverse career paths shaped by global inequalities.<br><br>Such changes acknowledge that career timelines and pathways vary globally and that rigid norms disadvantage many talented scientists in the Global South. Funding flexible

	diaspora engagement programs would harness the expertise of expatriate scientists in a more effective, inclusive, and empowering way. A growing number of programs and initiatives are beginning to support this vision, allowing researchers to maintain strong ties and collaborations with their home countries while addressing inequities in global science
Woma n	Give more visibility and credit to researchers that follow equitable, and fair practices.
Man	Authorship (credit) should go to the person(s) who do the work (the actual research and knowledge-writing ability) and not to the "lab", the "advisor", the "funder", or to a lesser extent to artist, data taker etc. -- those persons should be strongly acknowledged but not necessarily given authorship just because that is becoming the custom in large labs.
Man	If I could change one norm in global science, it would be the implicit rule that scientific leadership is defined by where funding and institutions are located rather than by intellectual contribution and local expertise.<br><br>In many international collaborations, researchers from well-resourced countries control funding, authorship, and decision-making, while scientists from the Global South are positioned primarily as data collectors or field facilitators. This structure undervalues local knowledge, limits capacity building, and reproduces inequities—even when collaborations are framed as "partnerships."<br><br>Changing this norm would mean requiring shared leadership by default: co–principal investigators across countries, transparent authorship agreements from the outset, and equitable control over data, specimens, and research agendas. Such a shift would not only be fairer, but also improve the quality and relevance of science, because research questions, interpretations, and applications would be grounded in both global theory and local context.<br><br>Equitable collaboration should not be treated as an ethical add-on; it should be a core criterion of scientific excellence.
Man	equal opportunities in publications and reduce cost
Woma n	if permits, fieldwork design and collection are part of the research then the providers of such needs to be invited for manuscript publication
Woma n	Funding acquisition is important but logistical resources and local expertise is equally important.
Woma n	For researchers from the south have more opportunities to do collaborative research and not just based in their local environment.
Woma n	More shaed and hoest collaboration not one sided
Woma n	The people in the north can't expect the people in the south to have the same number of papers or the same kind of publications. We don't have the same funding as them.
Man	I believe that the free movement of scientists, that is, the possibility of obtaining visas with reduced bureaucratic barriers, based on the academic and personal merit of the applicant, could become a policy that would greatly enhance scientific collaboration, particularly for researchers from the Global South.
Woma n	I would change the norm that implicitly ranks theoretical or conceptual contributions above contextual and field-based knowledge.<br><br>Recognizing local expertise, qualitative insights, and contextual understanding as central scientific contributions—especially in social sciences—would significantly rebalance collaborations and reduce extractive research practices.
Woma n	It is complicatd because , some barriers are mental in the case of Global soouth collaborators. Sometimes , it is easier collaborate with international researches that nationals. However , sometimes you felt used by internacional collaborators due to the nec4ssity of permits.
Woma n	To force having local partners when research is done in places different from the host institution. They could help balancing the potential power imbalances.
Woma n	That there sould be a journal system that allows people to submit their manuscripts on their native language, and this same system helps them to translate it in English, or something that helps to reduce the language bias.
Woma n	If I could change one rule in global science, I would require that any international collaboration include shared leadership and decision-making with institutions from the Global South. This means co-designing the research agenda, co-leading the project, and guaranteeing equal credit and authorship.

	Why? Because too often the South is included only as a field site or data provider, while the North controls the agenda, the resources, and the recognition. Shared leadership would shift collaborations from extractive to truly reciprocal, ensuring that knowledge and benefits do not flow in one direction only.
Man	Equal funding
Woman	The first thing I would do is change the high impact publications free of charge and that the works can be written in the native language of the first author.
Woman	The near-obligatory requirement that a good CV be measured in many cases by publications in high-impact journals, which greatly favors researchers from the north, means that mechanisms must also be created to assess the careers of researchers from the south who are of high quality but do not meet the standards of the north.
Woman	co-authority agreements previously agreed upon by institutional regulations
Man	First, we must open up the possibility of effective repatriation from other countries through repatriation plans approved by the relevant government institutions. Returning home cannot be seen as the only way to give back, because all it does is inflate a number (OECD index) without addressing the potential for improvement that exists abroad. What I mean is, the important thing is to bring back and develop knowledge to improve the quality of life for people in the country, not simply inflate a number.
Man	If you could change one rule or norm in global science to make collaborations more equitable, what would it be and why?<br><br>It would be good to have participation from the outset of the research proposal, involving leaders from all over and, according to the development of the proposal and the activities carried out by each research group, to rank the order of authorship.<br><br>In current collaborations, proposals are already made when ideas are shared, leaving participation in the global south only to provide data but not to lead research.
Woman	Scientist to scientist and the grant should be granted to the scientist and controlshould be done by an outstander that can citically follow the money fliw. This makes it easy to collaborate directly and effectively
Woman	Better financial support that award international collaboration . And Better chances for publications in high impact journal of researches in the global south.
Man	All researchers should need a local native collaborator in the country were the research is being done and secure all research permits necessary and funding an institutions should ask for those permits before research can be done/published
Woman	Not familiar with the rules, but in fact that could be one idea, make those rules widely known for members of each party so that not power relations are experienced
Woman	
Man	Give priority to applied research that allows the south to solve its development problems
Woman	I try to change the egoísm, so the new information must be show for the Richy.
Man	Maybe it could be that after a series of papers together (say 3), insitutions were forced to give double, crossed affiliation between partners.<br>The second is normalise co-first authorship and making it more visible/equitable
Woman	Decrease the influence of politics on science
Woman	Collaboration is perfect, but always involving at least a person from the place the material of the research is extracted: if the plant or animal is endemic of a country, a person frim there must be involved. Not having a specialist in this area, some could be formed!
Man	I don't know
Woman	The part of Becas Chile' contract that says that their researcher's are forced to move back to the country for around 8 years.
Man	I don't work in science
Man	Abolish 95% of the bureocracy
Man	The North should provide more financial assistance.
Woman	Try to work with experienced colleagues in the same topics, meaning collaborative work

Woman	None comes to mind. I feel it's a complex topic whose nuances I still don't fully understand
Woman	That papers sent by authors From the Global South are revised without predjudice.
Woman	Field work, data and specimen collection are equal valuable work
Woman	Even though I personally haven't faced problems in my collaborations, I do see one norm that could make a huge difference globally: shifting from "North-led by default" to shared leadership as the standard. Many projects still assume that the Global North should coordinate, manage, or "oversee" simply because of funding or institutional prestige. Changing this norm to require co-leadership with equal decision-making power, especially when the work addresses Global South populations, would immediately rebalance collaboration.
Woman	I propose to give more value to Social Sciences.
Woman	Have more conferences and networking events in the South, that would facilitate traveling and reduce costs for scientists from the poorest countries
Woman	Everyone that participates in the research should be a co-author and they should have a voice and vote. In the same way, everyone who collected the data should be responsible for the revision of the manuscript if they are a co-author.
Woman	I don't know, but there are a lot of things to change not only one rule. Is more the mentality of the people.
Man	The whole thing with indexing is wrong. Knowledge does not have to be indexed.
Woman	Researchers from the south should stop being just followers.
Man	Better access to funding and when funded, a .ore equitable participation of all the partners.
Woman	equal opportunities for all
Woman	La coordinación internacional para que los pensamientos de proyectos fueran dirigidos a la unión de los países en un bien común
Woman	colaboration from the beginning should be clear and the rules clearly establish! Not only being coauthors, but participating and deciding in every step of the project, that should be the main rule!
Man	Funding control
Man	Communication between regions
Woman	To turn obliged and open the detail of participation and contribution by each partner.
Man	que se valore segun el trabajo y aporte realizado
Man	The access to funding and research equipment. Using positive selection bias in order to promote research networks with the global south
Woman	Global funds shall find the way to get reliable local partners that act as supervisors/auditors to actually allow a better comprehensions of the souther partners. And avoid governmental contact/burocracy. Go directly to the scientific institution.
Woman	that when there are collaborations, the southern partner administrates the funding received.
Woman	Greater application of what has already been written?
Woman	If I could change one rule in global science, it would be to require that researchers from the Global South be included as equal partners—with shared decision-making power and authorship—from the start of any international project. This matters because equitable collaboration isn't just about funding or participation; it's about who defines the research questions and who benefits from the results. Ensuring equal leadership would make global science more fair, relevant, and impactful.
Man	Require the mandatory participation of authors and co-authors in the management of funds to increase opportunities for students from the global south.
Man	That any international scientific project involving human, biological, cultural, or financial resources from the Globall South must, by regulation, guarante genuine co-responsibility and equitable participation at all stages of the scientific process study design, decision-making, acces to funding, data analysis, lead authorship, and derived benefits because

	much of the current inequity does not stem solely from a lack of resources, but from the power asymmetry within collaborations
Man	It is difficult to say, because a rule could not be applied in all the countries and all the countries have different rules and regulations
Woman	ensuring research team members with presence and exposure in both the North and the South, trully exposed the realities of doing research in both workds, in both contexts
Man	Los tomadores de decisiones y financiamiento deberían volcar esfuerzos hacía las instituciones más pequeñas y que nunca han tenido la posibilidad de optar a los beneficios que otorguen, ,porque muchas convocatorias e información sólo llegan a las instituciones más grandes y tradicionales del país
Man	Researchers must be clear when assigning tasks and what do they mean regarding authorships
Man	Less bureaucracy and flexible curricula in the Global South institutions.
Man	I would like to reduce the political and economic barriers.
Man	N/A
Man	
Woman	More equal circulation of finances
Woman	I would start by changing the way peer-reviewed scientific publications are handled. It's important to mention that many scientists have been affected by this system, not only in the Global South. The publishing system has been criticized for sometimes lacking ethics and professionalism.
Man	One important source of inequality is the poor support given to Global South researchers by their own institutions, relative to the institutional support provided by Global North universities to their researchers. This includes inequalities of infrastructure, pay, and time allotted to research. If Global South governments could better nurture and support their scientists, they would be on a more equal footing.
Woman	The name of all the collaborators must be listed as principal, not only for the institution or person that put more money in the investigation
Woman	The costs — it is very expensive for a Latin American to be able to publish their articles in scientific journals that are prestigious and well-established in the First World.
Man	Sólo se logrará cuando el nivel de investigación sea equivalente e. g. Ecuador es la potencia mundial en acuicultura del camarón blanco, cualquier investigador extranjero tiene que hablar de igual a igual con los investigadores locales de I+D.
Man	To have more financial support to welcome professor frm north country in south research institute of university for long term period
Man	Reducing bureaucracy and extending research periods in Global South institutions would foster international collaboration. Currently, researchers from these institutions face cumbersome administrative processes to work abroad, and upon returning, are often burdened with numerous academic past duties that hinder them from continuing their research.
Man	Publishing research outputs in both English and the local languages
Woman	Deleted long legal documents for asking a permission to work with international partners
Man	
Man	
Woman	
Woman	
Woman	When working on a country different to the one where the researcher is based colleagues from that country must be involved always in all steps (field, days collection, analysis, writing, etc)
Woman	
Woman	Institutions could allow access to their publishing resources, such as open-access agreements, to any author of a manuscript, without making it contingent on being the first,

	last, or corresponding author because this can prevent leading authors from the south to get the recognition they deserve in pro to save money
Man	
Woman	
Woman	
Man	
Man	
Woman	
Woman	
Woman	
Woman	
Woman	
Man	
Woman	
Woman	

Q30 What is one thing researchers from the Global North often misunderstand about working with Global South partners? And vice versa?

Woman	Researchers from the North often assume that Global South partners lack expertise, tied to the misunderstanding that the South needs to be “helped". Researchers from the South think that partners from the North have full control on money or equitable collaboration; but the truth is that partners from the North are usually subjected to very strict funding rules and institutional policies that cannot be dismissed.
Man	Funding, funding, funding. Lack of funding equipment
Man	the lack of discipline
Man	One of the main misconceptions that researchers from the Global North often have when working with partners in the Global South is the assumption that we lack fluency in English or that our research does not have sufficient impact to be published — particularly when they are not familiar with our work.
Man	The Global North researcher may misinterpret the administrative sluggishness (or slowness) as a lack of efficiency or commitment on the part of the Global South partner.
Man	Non enough criteria to speak about it
Woman	One thing researchers from the Global North often misunderstand about working with Global South partners is the presence of extensive bureaucracy, which is sometimes mistaken as unwillingness or laziness to work. In reality, local administrative processes can be slow and complicated, influenced by limited resources and systemic challenges, not lack of effort. Additionally, the high local costs of conducting research and significant cultural differences affect collaboration dynamics and timelines.<br><br>Conversely, researchers from the Global South often misunderstand Global North partners as being more detached from local realities, expecting faster outputs without appreciating the contextual constraints of the South. Both sides need to recognize these structural and cultural differences to foster mutual respect and effective collaboration. Understanding that bureaucracy and costs are not indicators of motivation but challenges to be addressed jointly can help bridge these divides and make partnerships more equitable and productive

Woman	Unless they are familiar with it, they do not ackowledge the burden of bureacry and inefficiency. You could be the best, but the system in our countries is just difficult to navigate, specially for early researchers.
Man	North do not respect local knowledge as much as they should and South do not realize the types of pressure(s) Northerners face with regard to gaining grants, teaching responsibilities, etc.
Man	One common misunderstanding by researchers from the Global North is assuming that limitations in infrastructure or funding in the Global South reflect a lack of expertise or rigor. Conversely, researchers from the Global South sometimes misunderstand the extent to which Global North collaborators are constrained by their own institutional systems. Funding rules, ethics boards, legal frameworks, and publication pressures can limit flexibility, slow decision-making, and shape research priorities in ways that are not always visible. This can be misread as lack of commitment or unwillingness to share power, when in fact it reflects structural constraints rather than individual intent.<br><br>Recognizing that both sides operate within different—but equally real—systems of pressure is essential. Productive collaboration depends on making these constraints explicit, negotiating expectations early, and treating asymmetries not as personal failures but as structural challenges that require deliberate, shared solutions.
Man	They underestimate the capacities of the south peoples
Woman	that for us this is not a hobby, it is a job and the time we invest in the project is our working time that should be recognized
Woman	From the Global North underestimate our capacities and misunderstand our needs. From the Global South we assume all researchers from the North do what I described in the first sentence.
Woman	Probably that this is one helping the other. This is a partnership and peers work together
Woman	one. side have the money and dictate the rules and the other is force to stay at the same place (no progress at all)
Woman	The funding, the access to reagents
Man	As a researcher from the Global South, one of the most persistent misunderstandings I have encountered is the assumption of bias inherent in our academic backgrounds, which hinders meaningful discussion among peers. From the opposite perspective, yet rooted in the same underlying issue, ethnocentrism in the Global North often leads to the undervaluation of research outcomes when they originate from scholars in the Global South.
Woman	Researchers from the Global North often underestimate the complexity, rigor, and intellectual depth involved in producing contextual knowledge, particularly in fields related to work, health, and social conditions.<br><br>On the other hand, researchers from the Global South may underestimate how strongly academic norms, publication standards, and evaluation systems in the Global North shape research behavior, sometimes limiting flexibility even when there is goodwill.
Woman	The social situation ; burocracy, time-use , corruption , paper work.
Woman	The global north thinks the global South is less developed, while the south believes the north holds all the funding. The north has more funding but also much more buroacracy, making research far less creative and innovative.
Woman	I think that the Global North has to understand the historical and political perspective that science can have at the Global South, including their privileges.
Woman	One common misunderstanding from Global North researchers is assuming that Global South partners have limited knowledge or can only contribute as data collectors or local facilitators, not as thinkers who shape theories, methods, and agendas. This reduces our role to “fulfilling a requirement” instead of recognizing our intellectual contributions.<br><br>On the other side, Global South researchers may sometimes misunderstand the extent to which Northern institutions are shaped by their own constraints (funding rules, publication pressures, or institutional agendas), which can unintentionally reproduce asymmetries even when individuals have good intentions
Man	Way of working and lack of money
Woman	I don't know

Woman	They believe they should be the leaders in research and publications because they have the resources, the infrastructure, etc., and unfortunately many researchers in the south understand or accept these conditions for the same reasons.
Woman	Scientific Resilience: Conducting Research in the Global South with No Funding
Man	There are systemic structural differences that prevent truly equal work. As I mentioned earlier, the money invested in science is drastically different, and this affects the scientific world vertically, from the purchase of research materials like software to the hiring of people in various fields. I believe that those in the Global North must be more open to the context and conditions necessary to make publications and science itself accessible to people in the Global South if they truly want the planet's comprehensive development. Otherwise, we will perpetuate the structures that hinder global progress.
Man	What do researchers from the Global North tend to misunderstand about working with partners from the Global South? And vice versa?<br>They consider themselves to be better prepared, have more funding, and speak the language as their first language. And we have the research areas and sometimes the data.
Woman	The North always think the south is corupt , making my point in the earlier statement
Woman	They misunderstand that big problems we have with visa procedures and paperwork. Global South researcher misunderstand that researcher from GN can also be corrupt and have non appropriate practices and stereotypes on the collaborators.
Man	They do no value field work and do not want to comply with research permits necessary Institutions
Woman	Global North researchers are not aware of social dynamics or security issues in South countries and try to act on their own, but they should engage better with local communities. Global South researchers should know that they can work in equal conditions with same benefits as the northern partners
Woman	
Man	he most common thing is that those from the north obey private agendas and that there is little interest in a true cooperation with the south
Woman	Really i don’ t knowb
Man	Many times there is the idea that south only provides data/local experience, while the north has technology, money, and more talent. I think talent is equally distributed, but also, good science is not always about who has the faciest machine. And even then, we tend to forget that while the north has more funds, it tends to lack space. Something we did in my group is that we installed one of the fancy machines in South Africa, as they had more space. That built the capacity there for running specialised experiments, while we travelled when needed to run experiments alongside. It allowed us to use funds that were unreachable for them, but to install equipment that we would never had been able to operate in our lab space in London.
Woman	I think there are still issues because of cultural misunderstanding and different work style
Woman	Ih the Global North there are money and good equipments, but importants conserved Earth's biomes not always. Out the great North centers there are great brains, several without good equipments! When it is invested in South Institutions, good results are published
Man	Maybe they don't have much confidence in our institutions
Woman	Bourocracy?
Man	The diferent realities
Man	They want everybody to stick to their timeline. But in the south you have to improvise and be flexible because one day there is a power shortage, another a bus strike…
Man	I think it is that Global North partners think that paper work is smooth and less complicated as it is in the North.
Woman	Bureaucracy and deadlines
Woman	It seems that culturally, certain stigmas have developed. Other researchers from the Global South have made these stigmas evident
Woman	They don't understand the burocracy and insecurity pervasive in thirld World countries.

Woman	They assume we are not equal scientific partnera
Woman	Even though I've personally never had issues in my collaborations, I do notice some common misunderstandings on both sides. From the Global North, one misconception is assuming that limited resources mean limited expertise. Many Global South teams work with incredible precision and creativity because they're used to doing more with less. The depth of contextual knowledge is enormous, and sometimes that gets overlooked simply because it doesn't come packaged in the same institutional structure. From the Global South, there's sometimes an assumption that the North always has the "standard" or the "right" approach. That can make local teams hold back their insights, even when they are the ones who best understand the population, the politics, and the practical constraints on the ground.
Woman	The qualitative methodology is misunderstand in North and South Global.
Woman	North misunderstan: That we can just travel abroad and join them on equal footing at conferences and that we can easily access travel support, when in reality that kind of help is reserved to very poor countries ignoring that relatively wealthy countries in the south can still have a very underpaid research sector.<br>South misunderstand: That more often than not people will value your contribution and will have no problem in giving due credit (field dependent though)
Woman	The timing, they believe everything needs to be in a quick project scale, timeline, and things in the global bureaucracy due to bureaucracy and like a capacity take much longer
Woman	That in the South there are less financial opportunities and the work is performed in another way. And the North works fast because have millions to spend in one project. I believe south we are more open minded and North narrower.
Man	There are realities, that go into methods, that have to do with our contexts. Those should be taken into account.
Woman	Global North researchers think Global South researchers do not have the requisite competencies to lead research, and researchers from global south think they are taken advantage of.
Man	I think global north partners believe that global South collaborators are lazy, lack of knowledge and expertise.<br>While global South collaborators might assume that global north individuals want to steal resources. Both of them might depend on the context and might me wrong most of the times. It's important to set rules of collaboration a-priori and improve access to funding for global South partners
Woman	that we all have the same opportunities and abilities
Woman	Que los subestiman y los utilizan a su conveniencia
Woman	The capacity of Latam researchers! They confuse the institution's weakness in our countries with our knowledge or our leadership! But for that we can only blame our own governments! They only take advantages of the situation!
Man	Economic reality. Comfort v survival.
Man	Limited ability and experience
Woman	Different timing and work load, and the transversal intercultural diversity that some times leads to misunderstandings.
Man	desconozco
Man	The main research problems. It's quite difficult to agree on the research questions, due to opposite points of view.
Woman	We have limited funds but good ideas.
Woman	Northerns don´t get that we have highly skilled, creative and innovative people in the South, and that our governments support science in the best possible way (not common in all LATAM, but some at least), but it´s never enough due to limited resources. Southerns often dont get that despite working among scientists, some Northerns have a derogatory and biased way of seeing southern scientists, which impact in the project outcomes and outputs
Woman	It seems to me that in some cases they think we can take information away from them; and on our part, sometimes, that they only use us to get advantages and information and that they are not entirely honest about the work they are going to do.

Woman	One thing researchers from the Global North often misunderstand is that Global South partners don’t lack expertise—they often lack resources, not insight. As a result, Northern teams may overestimate the value of their own models and underestimate local knowledge.<br><br>Conversely, one thing Global South researchers sometimes misunderstand is that Northern partners may be unaware of local constraints not out of disregard, but simply because they’ve never worked in that context. Clear communication early on can prevent misinterpretations on both sides.
Man	Those in the north think that collaborators from the south should only be part of the research because otherwise they cannot have research permits, while those in the south see those from the north as providers of funds rather than as real cooperation.
Man	There is often a persistent perception that in the Global South we still live in primitive conditions, without technology or scientific capacity, for many, it is difficult to accept that in our countries we can conduct research at the same level as that carried out in the Global North, this is reflected in the constant distrust of our samples, data, and analyses in my case, I often have to submit complete databases, detailed analyses, and all available evidence simply to have the opportunity for a manuscript to be considered for review or acceptance although in theory uploading all this information is “optional" in practice it seems optional only for the North, while for the South it becomes practically an obligation.
Man	Some people from Global South see collaboration on terms of free trips and free stuff, instead to use the benefits; they travel o get training with no intention to use it. People from Global North do not understand the regulations in many countries, and they want free, expedit, and costless stuff from Global South, with no intention to offer training or couathorship to trained people (with PhD in the same field and highly proactive)
Woman	the gaps in most aspects are overlooked. Gaps in infrastructure, in research culture, in complying with committments, in dealing with deadlines. Acknowledging the gaps is critical for effectrive collaborations to ocurr
Man	Desconozco los términos de referencia por no tener la información no tengo un comentario
Man	Northern Researchers often mistake Southern researchers as less capable because they live less economically developed areas of the world. Southern researchers sometimes think Northern ones are where they are because of money only.
Man	Brazilian scholars tend to neglect Latin American colleagues as a potential partners.
Man	The northern ones sometimes have high expectations regarding knowledge capabilities about the southern ones, but often the differences in knowledge are huge.
Man	How to use data collected appropriately
Man	The efective time and the dedication that Global South researchers can have. Or the beurocracy in the south
Woman	North think in the south they might lack expertise, no: they just lack means. Southern see North as a treat: it is not, is an opportunity
Woman	Yes, many scientists from the Global North believe they won't find good partnerships in the Global South. This is because most scientists from the Global South don't publish in Q1 journals and therefore don't appear to be recognized globally. This is far from the truth. There are excellent researchers in the Global South with the academic capacity to produce high-quality publications, but there's a biased system that prioritizes studies conducted by scientists in the Global North or affiliated with world-renowned universities.
Man	Global North researchers often think of local collaborators as mere assistants to gather data, and often have no idea of the amount of valuable knowledge that Global South scientists could add to their project if given the chance. On the other hand, some Global South scientists believe that they deserve special treatment and disproportional credit just because they are local. This fosters "tokenism" and causes Global North scientists to under-value the capabilities of their Global South collaborators.
Woman	That they have the total control of Global South partners. On the other hand, the researchers of southern countries, even if the research is their idea, don’t take control over it because they think that they are subordinated by the person who invested more money.
Woman	Qualitative research is usually undervalued, even more so when it comes ‘from the South.’ It is as if scientific merit existed only in quantitative work, in statistics, and not in the interpretation of narratives—highly valuable ones—reflecting the perceptions of actors from the southern part of the continent.
Man	En nuestros países ha sido muy importante la investigación básica realizada en las últimas décadas del siglo pasado y que permitieron conocer en detalle nuestros recursos y ambiente. Actualmente, en un escenario de transformación digital, se genera una

	oportunidad para la I+D+i, dónde la investigación aplicada es protagonista. El modelo de las publicaciones de alto impacto no necesariamente aplica como la estrategia para resolver realmente nuestros problemas de investigación.
Man	Their is no monay to support publication initiative in south country. Financial support for experimental work is assymetric
Man	One thing researchers from the Global North often misunderstand is the severe time constraints faced by their Global South counterparts, due to excessive administrative controls and high teaching loads. Conversely, researchers from the Global South may not fully appreciate that access to well-funded facilities and resources in the Global North is also highly competitive and often difficult to secure.
Man	Global South-based collaborators are mostly field assistants
Woman	That we have long time just for research and some times we have less time for research and resources
Man	
Man	
Woman	
Woman	
Woman	
Woman	
Woman	
Man	
Woman	
Woman	
Man	
Man	
Woman	
Woman	
Woman	
Woman	
Woman	culture
Man	
Woman	Work-life balance, cultural difference s
Woman	

Q31 What do you think are the most meaningful actions—at the individual or institutional level—that could help build more equitable and respectful relationships in global scientific collaboration?

Woman	At the individual level, researchers should prioritise shared leadership from the beginning, value local expertise, and communicate transparently about authorship and resources. At the same time, institutions must support this by requiring co-led projects, offering funding that empowers both partners, enabling easier mobility, and enabling appropriate local infrastructure so all partners can participate fully in the scientific process.

Man	Scientific priorities. Research networks strengthening in the Global South.
Man	more incentives to research
Man	At both the individual and institutional level, one of the most significant actions would be to reduce bureaucracy. The sheer volume of documentation required to establish a partnership is genuinely frustrating and often slows down scientific progress.
Man	The creation of funded institutional programs that actively integrate the scientific diaspora from the country of origin into local teaching and research.
Man	Funding requeriments
Woman	Institutions should prioritize openness to international cooperation by establishing dedicated offices for global partnerships and allocating specific funds for equitable collaborations, such as direct grants to Global South institutions without requiring Northern leads. This ensures financial resources flow where research occurs, supporting local capacity and reducing power imbalances. Additional measures include redesigning funding to favor LMIC-driven agendas and creating joint monitoring frameworks to track equity throughout projects.<br><br>Individual Actions<br>Researchers can build respect by co-creating networks and projects from inception, valuing local expertise equally in authorship and decision-making. Actively learning about partners' cultural and systemic contexts prevents misunderstandings, while committing to long-term mentorship fosters mutual growth. These steps promote reciprocity and shift from extractive to collaborative models.
Woman	Talk openly about these issues. I'd love to see some courses on this issues on my main institution in Europe. However, most of them are directed to the social sciences side but forget about other groups that travel frequently to other countries for natural sciences. - Make it mandatory but easly accesible to collaborate with local institutions.
Man	North still needs to provide opportunities to South in the field and lab, for travel and education, publishing responsibilities.
Man	To build more equitable and respectful relationships in global science, individuals should prioritize mutual respect, shared leadership, and transparent discussions on authorship and data ownership. Institutions can foster equity by ensuring funding supports Global South partners directly, valuing local expertise, and requiring fair collaboration structures. Ultimately, shifting the definition of scientific excellence to include equity will make these practices the norm.
Man	Fostering equitable global scientific collaborations requires individuals to practice humility and transparent communication, while institutions must adopt fair funding models, shared ownership policies, and robust capacity-building initiatives.
Woman	Active communication. It is not ok to share the paper when is done, even if you name is included. We should be part of the entire research process
Woman	Facilitate exchange opportunities.
Woman	For researchers to be able to travel, meet other researchers, being able to network and share
Woman	Start by accepting that scientist of the south have the same rights to evolve give them just the same tools and lets work on the same level
Woman	To see as equal researchers even do we don’t have the same CV as them
Man	At the individual level, researchers in developing countries must continue to ground their work in the ethical principles and scientific rigor that the discipline demands, as a principled response to prejudice. At the institutional level, it is essential to secure funding mechanisms that ensure equitable collaboration between peers from the Global North and the Global South. In this context, equity encompasses all stages of the research process, from government support to fieldwork and laboratory-based activities.
Woman	At the individual level, taking time to listen, acknowledging positionality, and being transparent about constraints and expectations are essential first steps.<br><br>At the institutional level, training on power dynamics in research, clearer frameworks for equitable collaboration, and greater recognition of qualitative and applied research—such as work on quality of working conditions—would help create more balanced and respectful partnerships.
Woman	Clear communicationn

Woman	Respect
Woman	I think keeping a bidirectional communication, and having a conversation with an open mind about their personal experiences that have influenced in their research experience can be the key component to change collaborations.
Woman	Meaningful actions start by acknowledging power imbalances. Individually, this means co-designing projects and sharing credit fairly. Institutionally, it requires funding and rules that support shared leadership and long-term partnerships defined with, not for, the Global South.
Man	Faciliting physical meetings and travel
Woman	perhaps sign agreements clearly specifying roles, costs and profits for each party. And make them comply.
Woman	Collaborative agreements must be drawn up that value and protect the knowledge of researchers from the south and ensure that this knowledge is respected.
Woman	ethical rules at the institutional level
Man	Promote the departure of researchers from the southern hemisphere to build networks in the northern hemisphere, with a plan for compensation from the centers in the north, through the search for scholarships, funds and opportunities for researchers.
Man	What do you think are the most significant actions, at the individual or institutional level, that could help build more equitable and respectful relationships in global scientific collaboration?<br>Create funds to finance research projects and unique publications with equitable global participation with two leaders (one from the South and one from the North). Promote scientific journals from the global South so that they are rated and attractive like those from the global North.
Woman	Just talk directly to the depatment and the scientist , this works best since you will have direct contact and problems are solved in a short time span
Woman	The mean actions will be related to work in equity and negociate a 50/50 collaboration that work in both ways.
Man	Institutions should be more aware of what your researchers are doing and have clear rules about money going to the south, collaborations and research permits
Woman	Training and engaging in the ‘opposite’ environment, so that members of each party feel familiar with the working way in each region
Woman	
Man	At the outset, mention the national agendas and interests of research and utilitarian to the interests of local development
Woman	I agree
Man	More grants for collaboration, maybe even request journals to have special issues where only collaboration science north-south/south-south can be published
Woman	Make visa rules less strict, help researches from abroad with finding apartment, finance mobility better
Woman	Investment to send younger students to important centers as part of theirs formations is the first action to invest
Man	To show them that we are as good as they are or consider to be
Woman	Respect, and strict policies about how to do field work and sample collection in order to don't fall into unethical practices.
Man	Cooperation and funding
Man	Abolish diploma revalidations and turn it all digital
Man	Developing Intercultural Competence, Willingness to contribute financial resources to advance endeavors,
Woman	Sharing data and knowledge, delegating tasks to the colleagues from the South
Woman	To maintain solid ethical and moral standards
Woman	Maybe not assuming that the scientists From the Global South have access to the same resources and opportunities as those from the Global North.
Woman	

Woman	From what I’ve seen, the most meaningful actions happen on two levels. Individually, the biggest shifts come from simple habits: listening before proposing solutions, giving credit fairly, and treating partners as equals rather than extensions of a project. When people show up with curiosity, transparency, and respect for local expertise, everything flows better. Institutionally, what really changes the landscape are structures that support equity instead of just talking about it. Things like shared leadership requirements, long-term funding instead of one-year extractive projects, and real investment in local capacity and data infrastructure.
Woman	Notth Global shouldnt have prejuices.
Woman	Researchers from the north should collaborate with the sith in more than just accessing data, they could direct funding to invite students to their facilities and train them
Woman	Strong memorandum of understanding and data sharing agreements to protect local scientist
Woman	Researchers should work together between north and south and stop to compete each other. Specifically in the same field. More integration, labs multicultural, more international exchanges, more political agreements. There are a lot of work, and people need to change.
Man	Resisting the worldwide discourse about indexing and supposedly "scientific" work.
Woman	Come to the table, discuss issues, assign tasks with KPIs, report at agreed times.
Man	Treat with respect regardless the origin of people, be aware of the structural inequalities, and participate actively to make participation inclusive for all.
Woman	At an individual or institutional level, we must ensure that the rights we all have are respected.
Woman	A nivel individual y colectivo sería el aporte de todos, con principios éticos dando lo mejor de cada persona o colectivo en el criterio de la verdad
Woman	Get ours house in order first!! And get smart in negotiating everything clear from the beginning!
Man	Funders
Man	Mobility between colleagues, training
Woman	Building capacity for Global South and Global North to recognize and valuate its contributions under an equity, diversity and inclusive perspective, which can be strengthened under UN and other IGOs frames.
Man	Mas globalizadas
Man	Thinking about a science that look further than the trending research problems.
Woman	
Woman	Having committed and proactive scientists from the south working in the north to bypass and demonstrate our capabilities, scientific rigor, and integrity. This can also broaden the path for future southern scientists, that wont need to climb the everest to collaborate at international level because at least a small path was build
Woman	At an institutional level, improve agreements; at a personal level, improve exchanges or communication networks between researchers to facilitate alliances.
Woman	The most meaningful actions are those that shift power and build trust. At the individual level, this means sharing credit fairly, listening to local priorities, and being transparent about expectations and funding. At the institutional level, it means creating funding structures that require co-leadership, supporting long-term partnerships instead of short-term extractive projects, and investing in research capacity in the Global South. Together, these actions make collaboration more equitable, respectful, and sustainable.
Man	If science had more local funding, we wouldn't have to depend so much on seeking participation to carry out our research ideas, we would need more high-tech laboratories to avoid transporting samples, etc.
Man	Mandatory co-responsibility policies, ensuring equitable participation of the Global South in study design, lead authorship, decision-making, access to resources and benefits, promoting fair funding and transparent distribution, and avoiding models in which the Global North concentrates financial control while the Global South is limited to merely carrying out fieldwork.
Man	Active collaboration, but including proactive staff. Everywhere in Global South there are people who want to be included in High Class papers, but without improving or adding

	meaningfull actions on it. I have seen people "parasiting" papers or projects. Then, in future projects, Global North people do not want to include local people for such reasons
Woman	the North researchers being exposed to the precarousness experience in the South and those researchers in the South inhance understanding of good practices. That is why the science diasporas are key to address the gaps
Man	Considerar las condiciones de acuerdo al tipo de institución flexibilizando algún requisito
Man	Individuals
Man	More open access journals.
Man	Many southern researchers do not speak English. Institutions must work more on this, as English is the universal language for science.
Man	Pushing back against exploitative authorship practices
Man	
Woman	More inclusion of southern institutuions. Regional government should give more funds
Woman	There should be more conferences or workshops organized by academics from the Global South, within the Global South itself, to strengthen ties in the region. Often, even scientists from the Global South have little contact, and consequently, research networks are lost. People from the Global North should also be included to send the correct message that the Global South has capacity, scientific networks, and networks that intend to continue growing.
Man	More exposure to each other, and improved socioeconomic support for science (unfortunately at this moment in time, both the Global North and the Global South are treating their science and scientists very poorly).
Woman	The researchers should understand that in a work like this there is no superiority. The institution should promote this idea
Woman	Possibly, one meaningful action at the institutional level is financial support. Unlike at the university where I work, where you have to make the financial investment yourself to see if your text gets published in a prestigious journal—and if it is published, they might reimburse you, but if not, they don't—institutions should provide direct economic backing.
Man	Promover aquellas líneas de investigación en que somos más fuertes, generando un respeto internacional en esas áreas. Implementar un modelo de triple hélice e innovación colaborativa + responsabilidad social coo mecanismos de transición a la I 5.0
Man	Take into account the different level of economic conditions from both country
Man	Promote brief mutual exchanges and request open, globally confidential projects that are double-blind evaluated.
Man	
Woman	Open borders, free speech and collaboration with protection of the scientific production
Man	
Man	
Woman	
Woman	
Woman	
Woman	
Woman	
Man	
Woman	
Woman	

Man	
Man	
Woma n	
Woma n	
Woma n	
Woma n	
Woma n	respect
Man	
Woma n	
Woma n	

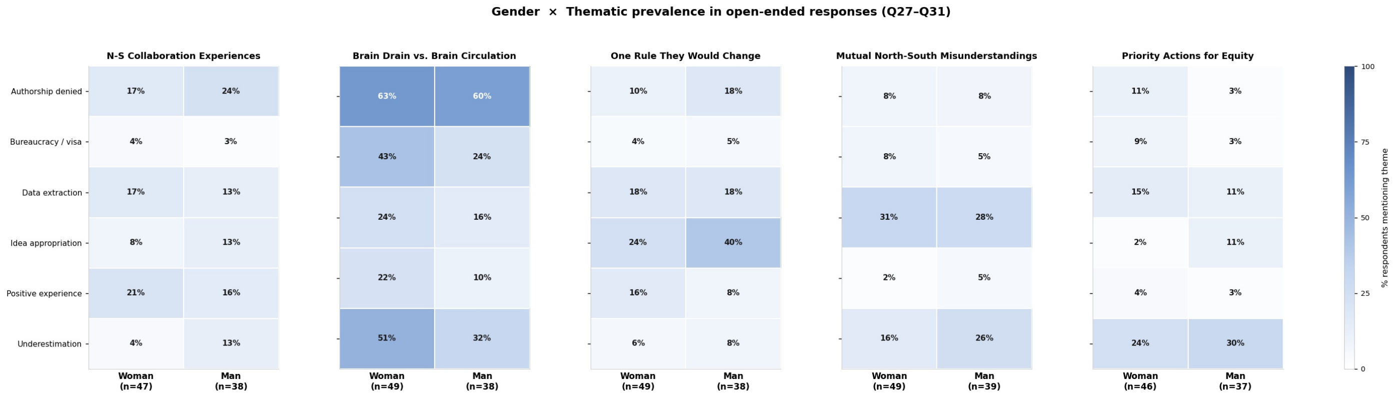

Note. Values = % of respondents within each group whose response contained at least one keyword associated with the theme. Only respondents providing a non-empty answer are included in each question's denominator.
